# Anti‐MicroRNA‐21 Oligonucleotide Loaded Spermine‐Modified Acetalated Dextran Nanoparticles for B1 Receptor‐Targeted Gene Therapy and Antiangiogenesis Therapy

**DOI:** 10.1002/advs.202103812

**Published:** 2021-12-22

**Authors:** Tao Zheng, Wentao Wang, Mohsen Mohammadniaei, Jon Ashley, Ming Zhang, Ninglin Zhou, Jian Shen, Yi Sun

**Affiliations:** ^1^ Department of Health Technology Technical University of Denmark Kongens Lyngby DK‐2800 Denmark; ^2^ Jiangsu Collaborative Innovation Center for Biomedical Functional Materials School of Chemistry and Materials Science Nanjing Normal University Nanjing 210023 P. R. China

**Keywords:** antiangiogenesis therapy, anti‐microRNA‐21 oligonucleotide, gene therapy, glioblastoma multiforme, targeted delivery

## Abstract

The use of nanoparticles (NPs) to deliver small inhibiting microRNAs (miRNAs) has shown great promise for treating cancer. However, constructing a miRNA delivery system that targets brain cancers, such as glioblastoma multiforme (GBM), remains technically challenging due to the existence of the blood‐tumor barrier (BTB). In this work, a novel targeted antisense miRNA‐21 oligonucleotide (ATMO‐21) delivery system is developed for GBM treatment. Bradykinin ligand agonist‐decorated spermine‐modified acetalated dextran NPs (SpAcDex NPs) could temporarily open the BTB by activating G‐protein‐coupled receptors that are expressed in tumor blood vessels and tumor cells, which increase transportation to and accumulation in tumor sites. ATMO‐21 achieves high loading in the SpAcDex NPs (over 90%) and undergoes gradual controlled release with the degradation of the NPs in acidic lysosomal compartments. This allows for cell apoptosis and inhibition of the expression of vascular endothelial growth factor by downregulating hypoxia‐inducible factor (HIF‐1*α*) protein. An in vivo orthotopic U87MG glioma model confirms that the released ATMO‐21 shows significant therapeutic efficacy in inhibiting tumor growth and angiogenesis, demonstrating that agonist‐modified SpAcDex NPs represent a promising strategy for GBM treatment combining targeted gene therapy and antiangiogenic therapy.

## Graphical abstract

In this work, a novel targeted antisense miRNA‐21 oligonucleotide delivery system for glioblastoma (GBM) treatment is developed, achieving in inhibiting tumor growth and angiogenesis, demonstrating that agonist‐modified SpAcDex nanoparticles represent a promising strategy for GBM treatment.

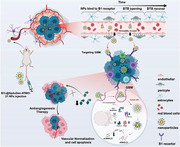

## 1 Introduction

Currently, glioblastoma (GBM) is recognized as one of the most challenging tumors to cure. It is highly heterogeneous and aggressive, characterized by uncontrolled cellular proliferation, diffuse infiltration throughout the brain, extensive angiogenesis, resistance to apoptosis, and the development of necrosis.^[^ ^1^ ^]^ Although we are increasing our understanding of the mechanisms of GBM tumors, patient life expectancy has not been improved.^[^ ^2^, ^3^, ^4^ ^]^ In recent years, the emergence of gene therapy for cancer treatment has attracted a lot of attention. Gene therapy has significant therapeutic potential to treat various cancers, as it can target the specific gene sequence of diseases with high selectivity.^[^ ^5^, ^6^, ^7^, ^8^ ^]^ MiRNA is an interesting type of gene regulator that has been extensively studied by scientists.^[^ ^9^, ^10^ ^]^ MiRNAs are small noncoding RNAs of 18 to 25 nucleotides that regulate protein expression by targeting mRNAs to either enhance or inhibit translation. MiRNA‐21 is strongly overexpressed in the malignant human brain and can boost cell proliferation through inhibiting programmed cell death protein 4 (PDCD 4) and phosphatase tensin homolog (PTEN) or by targeting several signaling pathways that induce cell apoptosis.^[^ ^11^, ^12^ ^]^ High‐throughput screening studies have reported that miRNA‐21 plays a vital role in stimulating angiogenesis by targeting angiogenesis‐related genes.^[^ ^9^ ^]^ Therefore, we studied the effect of antisense miRNA‐21 oligonucleotide (ATMO‐21) on the growth of blood vessels in the brain tumor microenvironment.

To date, only ten oligonucleotide drugs have been approved by the Food and Drug Administration for clinical treatment.^[^ ^13^ ^]^ The major hindrances preventing their widespread application include low delivery efficiency to the tumor site, off‐target interactions, and low stability after reaction with serum and nuclease in the extracellular environment. Recently, nanotechnology has greatly facilitated the delivery of oligonucleotide drugs.^[^ ^14^, ^15^ ^]^ Various biocompatible polymeric materials have emerged as feasible ways to deliver miRNA‐based gene therapies.^[^ ^16^, ^17^, ^18^, ^19^ ^]^ Although some delivery systems have achieved satisfactory results in tumors, showing obvious inhibition efficiency both in vitro and in vivo compared with naked miRNA. However, this gene delivery system often have a low encapsulation rate and short blood circulation time.^[^ ^20^, ^21^ ^]^ To increase both delivery and loading efficiency, we developed SpAcDex, a polymer with excellent biocompatibility and biodegradability, to encapsulate ATMO‐21. The positive charges of the polymer together with the double emulsion synthesis method significantly improved the encapsulation efficiency of ATMO‐21 to exceed 90%, achieving the highest encapsulation efficiency of ATMO‐21 so far.^[^ ^22^, ^23^ ^]^ Moreover, as a pH‐sensitive polymer, SpAcDex degrades faster in tumor tissues (pH 6.8) or lysosomal compartments (pH ≈ 5.0–5.5) than in physiological conditions (pH 7.4), which minimizes off‐target effects and reduces the metabolic time of NPs in the blood circulation.^[^ ^24^, ^25^ ^]^

The delivery of genes to the brain is made complex by the natural obstacles in the brain, including the BTB, which limits penetration of the NPs into the tumor site, reducing the delivery efficiency of oligonucleotide drugs.^[^ ^26^, ^27^, ^28^ ^]^ Therefore, the systematic design of vectors that can overcome these delivery challenges is necessary for the successful targeted delivery of miRNA into the tumor site of the brain. Some conventional ways to open the BTB include physical methods (photothermal effects, ultrasound effects, etc. that have irreversible damage to the brain), and some nano‐delivery systems modified by peptides or proteins that can target the BTB. However, these methods often exhibit low delivery efficiency. Adjusting the permeability of the BTB provides a powerful strategy for brain tumor‐targeted delivery, which can potentially increase the efficiency of gene delivery and further improve the therapeutic effect in GBM treatment.^[^ ^29^ ^]^ In our previous work, we found that kinin B1 receptor‐modified NPs could effectively traverse the BTB and target tumor cells. Kinin peptides, a type of natural agonist, can regulate the permeability of vessels by activating G‐protein‐coupled receptors, including the B1 receptor and B2 receptor.^[^ ^30^ ^]^ Compared with B2 receptors, which are expressed in numerous tissues, B1 receptors are barely expressed in physiological tissues except in the central nervous system.^[^ ^31^ ^]^ In addition, GBM‐derived cytokines induce inflammation regions that maintain tumor proliferation and angiogenesis, triggering B1 receptor activity and expression in brain tumor microvascular systems.^[^ ^32^ ^]^ Hence, we projected that increased permeability of the BTB occurs when B1 receptor ligand (B1L) binds to its receptor on the blood vessels in GBM areas. Accordingly, we functionalized ATMO‐21‐loaded NPs with the B1L ligands and investigated their safety and the efficiency of their gene delivery to brain tumors. Based on the knowledge that GBM is highly vascular and expresses high levels of the angiogenic mediator vascular endothelial growth factor (VEGF), there have been several clinical works on systemically administering anti‐VEGF monoclonal antibodies.^[^ ^33^ ^]^ However, the result is not as expected due to the natural obstacles of the BTB. Therefore, we projected that an oligonucleotide delivery system overcoming the BTB, and allowing for local and persistent delivery of ATMO‐21 to the central nervous system would present an efficient strategy to treat GBM.

**Figure 1:**
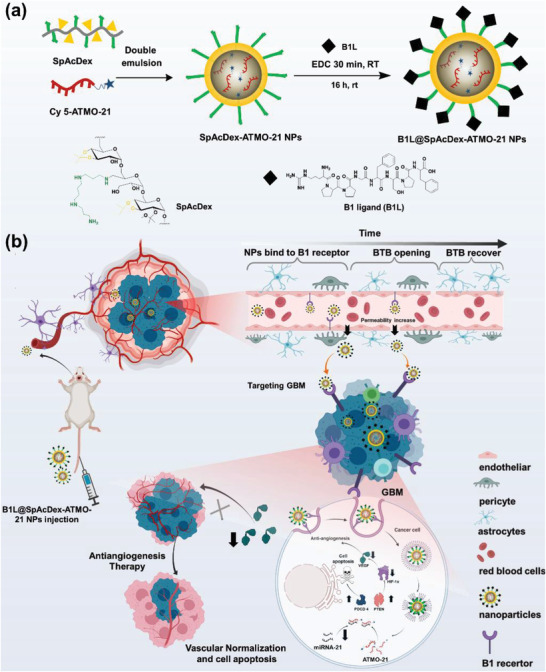
a) Schematic of the synthesis of B1L@SpAcDex‐ATMO‐21 NPs. b) The treatment of brain tumors using miRNA‐21‐inhibiting oligonucleotides and antiangiogenic therapy. Modified icons from BioRender.com.

In this work, we designed a GBM‐targeting anti‐miRNA‐21 delivery system composed of (1) Cy5‐modified ATMO‐21 oligonucleotides, (2) a pH‐responsive polymer of SpAcDex to formulate NPs, and (3) (des–Arg^9^)–bradykinin as a B1 receptor agonist to modify the surface of NPs. SpAcDex has a highly positive charge and therefore binds efficiently to ATMO‐21 via electrostatic interactions, achieving a high loading efficiency (over 90%) of ATMO‐21 compared with traditional vectors, including polyethylenimine (PEI) and liposomes (**Figure** **1**a). In addition, the SpAcDex‐based delivery platform is sensitive to acidic conditions, which is the desired characteristic for tumor‐specific release.^[^ ^34^ ^]^ More importantly, the spermine‐modified dextran produced by the hydrolysis of SpAcDex NPs may be further metabolized by intracellular enzymes.^[^ ^35^, ^36^ ^]^ The B1L‐decorated NPs exhibited improved penetration of the BTB in vivo and excellent targeting ability for GBM cells via flow cytometry analysis. When NPs are taken up by GBM tumor cells, ATMO‐21 is released, which promotes the apoptosis of GBM cells by upregulating the expression of PDCD 4. In addition, the upregulation of PTEN, downregulation of HIF‐1*α*, and finally decreased VEGF expression, promote the normalization of blood vessels in the tumor microenvironment. Based on tumor slice staining after treatment with NPs, we found that the reduction of blood vessels in the tumor microenvironment and pericytes, demonstrated the antiangiogenic therapy. The anti‐microRNA‐21 oligonucleotide drug delivery system with accurate and controllable release performance holds promise for the treatment of GBM with improved therapeutic efficiency and reduced side effects (Figure 1b).

## 2 Results and Discussion

### 2.1 Design, Fabrication, and Characterization

SpAcDex, an intrinsic adjuvant with excellent biocompatibility and acid sensitivity, was used to fabricate NPs. The synthesis and characterization (^1^HNMR spectrum) of SpAcDex are shown in Figures S1–S4, Supporting Information. In a classical method, natural dextran obtained from *leuconostoc mesenteroides* was first oxidized to generate partially oxidized dextran. Then, acetalation‐oxidized dextran was synthesized. The produced AcDex was finally modified with spermine through the Borch reductive amination method to obtain SpAcDex. SpAcDex NPs encapsulating ATMO‐21 were prepared by a water/oil/water (double emulsion) method (Figure S5, Supporting Information). Cy5‐conjugated ATMO‐21 was incorporated into the aqueous phase in this method, and SpAcDex was dissolved in dichloromethane as an organic phase. Polyvinyl alcohol was used to stabilize the high‐energy sonication process. The resulting double emulsion solution was then subjected to 3 h of evaporation to remove the solvent. Empty NPs (without ATMO‐21) were fabricated according to the above similar conditions. The ATMO‐21‐encapsulated NPs were further functionalized with B1L through traditional crosslinking chemistry.^[^ ^37^, ^38^ ^]^

**Figure 2:**
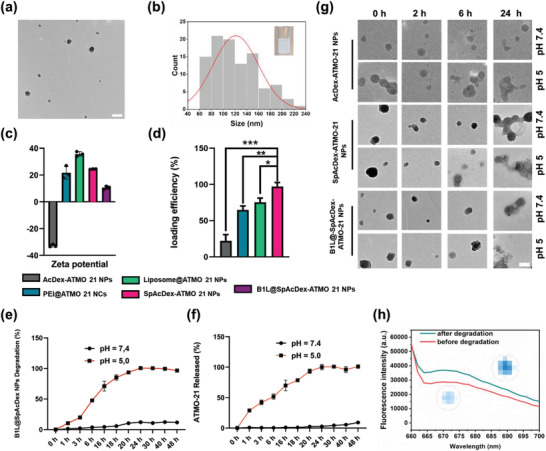
Characterization and release behaviors of ATMO‐21 from as‐fabricated NPs (AcDex‐ATMO‐21 NPs, SpAcDex‐ATMO‐21 NPs, and B1L@SpAcDex‐ATMO‐21 NPs). a) TEM images of B1L@SpAcDex‐ATMO‐21 NPs. Scale bar, 200 nm. b) Size of B1L@SpAcDex‐ATMO‐21 NPs measured by TEM; the inserted photograph is a photograph of B1L@SpAcDex‐ATMO‐21 NP solution. c) Zeta potentials of as‐fabricated NPs. Data are presented as means ± SD (*n* = 3). d) ATMO‐21 loading efficiency in NPs. Data are presented as mean ± SD (*n* = 3, one‐way ANOVA, *p < 0.05, **p < 0.01, ***p < 0.001). e) Degradation of B1L@SpAcDex‐ATMO‐21 NPs (without ATMO‐21) incubated at pH 7.4 and pH 5. Data are presented as means ± SD (*n* = 3). f) Release of ATMO‐21 from B1L@SpAcDex‐ATMO‐21 NPs incubated at pH 7.4 and pH 5. Data are presented as means ± SD (*n* = 3). g) TEM images of morphology monitoring of AcDex‐ATMO‐21 NPs, SpAcDex‐ATMO‐21 NPs, and B1L@SpAcDex‐ATMO‐21 NPs at different time points (0, 2 h, 6 h, and 24 h) of incubation in simulated cell conditions at pH 7.4 and pH 5. Scale bar, 100 nm. h) Fluorescence spectra of B1L@SpAcDex‐ATMO‐21 NP solutions incubated at pH 7.4 and pH 5.

We then investigated the size distribution and morphology of the as‐fabricated NPs using transmission electron microscopy (TEM) imaging (**Figure** **2**a,b). The TEM images showed that B1L@SpAcDex‐ATMO‐21 NPs had a homogenous size distribution (135 ± 26 nm). However, a larger size (170.4 ± 30.5) was obtained from dynamic light scattering (DLS) (Figure S6, Supporting Information) because TEM measures the NPs in a dry state, while DLS measures them in an aqueous environment. As shown in Figure 2c,d and Figure S7, Supporting Information, we selected PEI25K and liposome platforms, which have been used intensively for gene delivery, as a benchmark to evaluate gene loading efficiency and delivery efficiency SpAcDex. We found that the zeta potential of SpAcDex‐ATMO‐21 NPs increased to +24.6 mV with a good polydispersity index (PDI) of 0.12 compared to AcDex‐ATMO‐21 NPs (‐33.2 mV, 0.32 PDI) due to the amine groups from the spermine functionalization. The PEI@ATMO‐21 nanocomplex (NCs) and Liposome@ATMO‐21 NPs had zeta potentials of +25.3 mV (59.65 nm, 0.13 PDI) and +35.6 mV (175.6 nm, 0.53 PDI), respectively. However, NPs with a high cationic surface charge (over +20.0 mV) can easily bind to serum proteins, further inducing aggregation problems under physical conditions. After modification with B1L, the surface charge decreased to + 9.3 mV with a PDI of 0.45, which prolonged the circulation time in the blood vessels and improved stability in the complex biological environment (Figure 1c).

To investigate ATMO‐21 loading properties, four types of loading substrates, PEI25k, liposomes, AcDEX, and SpAcDex, were prepared to encapsulate ATMO‐21 (wt/wt, 1.0 µg mg^−1^). Following preparation, the loading efficiency of ATMO‐21 into the NPs was quantified by using a NanoDrop (Figure 2d). Generally, the as‐fabricated NPs or NCs encapsulating ATMO‐21 were dissolved in acetate buffer at pH 5.0 and further incubated in a 37 °C shaker. The supernatant was obtained by centrifugation at 14 800 rpm after 24 h of gentle agitation. Loadings of up to 1.0 µg of ATMO‐21 per mg of NPs were achieved, and the loading efficiency was above 80% for both PEI 25K (85%) and SpAcDex (nearly 100%) substrates, while the loading efficiency of the AcDex substrate reached only approximately 20%. Based on these results, the spermine‐functionalizing surface of AcDex will facilitate the generation of NPs with high loading of ATMO‐21. In this way, we found that the NPs obtained by double emulsion technology could also produce NPs with high gene loading efficiency compared with electrostatic interactions (PEI@ATMO‐21 NCs, Liposome@ATMO‐21 NPs).

### 2.2 pH‐Dependent Degradation and ATMO‐21 Release

Tuning the release rate of the encapsulated cargo has become the critical factor for commonly used gene delivery systems.^[^ ^39^, ^40^ ^]^ To evaluate the pH‐dependent degradation properties of NPs, we incubated NPs (AcDex NPs, SpAcDex NPs, and B1L@SpAcDex NPs) without ATMO‐21 in acetate buffer (pH 5.0) to simulate the intracellular microenvironment and also incubated them under normal physiological conditions (pH 7.4). From Figure 2e, we can observe the degradation behavior of B1L@SpAcDex NPs, and only slight changes in their integrity occurred at pH 7.4. Similar degradation curves of AcDex NPs and SpAcDex NPs are shown in Figure S8, Supporting Information. However, a clearly faster degradation rate for these NPs occurred at pH 5. The data are in line with the results of the ATMO‐21 release performance in Figure 2f. Then, ATMO‐21 was packaged into AcDex NPs, SpAcDex NPs, and B1@SpAcDex NPs to assess the pH‐dependent release from NPs, and the same conditions were used as described above. As expected, NPs showed continuous release during the first 24 h when incubated in pH 5.0 buffer, while less ATMO‐21 was detected at pH 7.4 after 48 h of incubation, which indicates a slight release of ATMO‐21. TEM images (Figure 2g) of the morphology of the as‐fabricated NPs and UV–vis spectra (Figure S9, Supporting Information) and fluorescence spectra of B1L@SpAcDex‐ATMO‐21 NPs at different time points of incubation at pH 7.4 and pH 5 (Figure 2h) further proved the controllable release of ATMO‐21 from this kind of NP. Most importantly, the miRNA cargo will be released only into the acidic subcellular compartments of the cancer cells of the tumor site, which is ideal for intravenous administration and makes this type of polymer a good candidate for continuous gene delivery vectors in the future.

### 2.3 In Vitro Evaluation of ATMO‐21 Delivery

**Figure 3:**
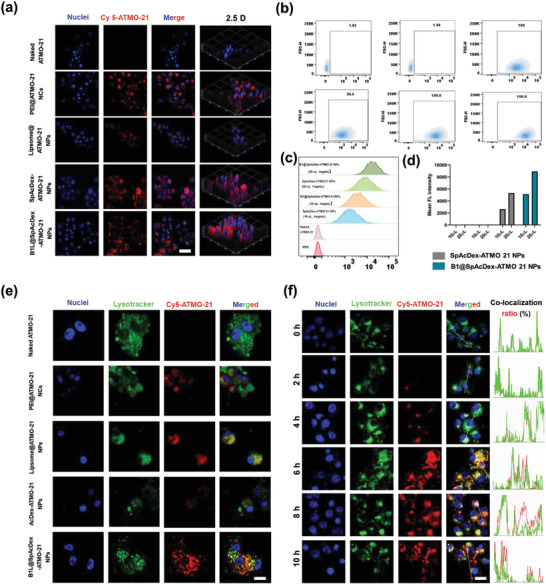
In vitro cellular uptake, target ability, and endosomal escape of B1L@SpAcDex‐ATMO‐21 NPs (red fluorescence) measured by CLSM and flow cytometry. a) CLSM images of cell uptake within U87MG cells incubated with naked ATMO‐21, PEI@ATMO‐21 NCs, liposome@ATMO‐21 NPs, SpAcDex‐ATMO‐21 NPs, and B1L@SpAcDex‐ATMO‐21 NPs. Scale bar, 20 µm b) Flow cytometry analysis of the delivery efficiency of B1L@SpAcDex‐ATMO‐21 NPs. c) Analysis of the targeting ability of B1L‐modified NPs. d) Mean fluorescence intensities of SpAcDex‐ATMO‐21 NP‐ and B1L@SpAcDex‐ATMO‐21 NP‐treated U87MG cells after 10 h of incubation. e) CLSM images of endosomal escape of as‐fabricated NPs (naked ATMO‐21, PEI@ATMO‐21 NCs, Liposome@ATMO‐21 NPs, SpAcDex‐ATMO‐21 NPs, and B1L@SpAcDex‐ATMO‐21 NPs) measured by CLSM. Scale bar, 10 µm. f) pH‐responsive endo/lysosomal escape of B1L@SpAcDex‐ATMO‐21 NPs. The corresponding colocalization fluorescence intensity and the colocalization ratios between NPs (red fluorescence) and endososomes (green fluorescence) in U87MG cells when incubated with B1L@SpAcDex‐ATMO‐21 NPs for 10 h measured by CLSM. Scale bar, 10 µm.

To investigate the ATMO‐21 delivery performance of B1L@SpAcDex NPs compared to PEI25k, liposomes, and SpAcDex NPs, ATMO‐21 labeled with the fluorescent molecule Cy5 was encapsulated into polymers. The delivery efficiency and targeting ability of B1L‐modified NPs were evaluated by confocal laser scanning microscopy (CLSM) and flow cytometry after incubation with U87MG cells (Human glioma cells), C6 cells (Rat glioma cells), and human astrocytes for 24 h. The NPs were visualized in red (**Figure** **3**a, Figure S10, Supporting Information). Together with the flow cytometry and CLSM results in Figure S11, Supporting Information, both the SpAcDex‐ and B1L@SpAcDex‐encapsulated ATMO‐21 groups exhibited strong fluorescence intensity signals and high delivery performance, verifying that SpAcDex‐producing NPs were taken up by U87MG cells and C6 cells most efficiently. In contrast, the uptake of B1L‐modified NPs by human astrocytes (Figure S12, Supporting Information) was lower than that of NPs without modification, which is in line with the quantitative analysis via flow cytometry in Figure S13, Supporting Information, suggesting that B1L‐decorated NPs can better target brain cancer cells, thereby minimizing damage to normal cells. At the same time, a gradual signal between the lysosome and NPs could be observed in B1L@SpAcDex‐ATMO‐21 NP‐treated cells, showing that more efficient endosomal escape occurred in this treatment group (Figure 3e, Figure S14, Supporting Information). The physiological toxicity of PEI25K makes it unsuitable as a gene delivery vector even though it possesses a high gene delivery efficiency.

The cellular uptake of ATMO‐21 was also quantitatively analyzed by flow cytometry (Figure 3b) to evaluate the targetability of B1L in U87MG cells. The results are in line with the CLSM results for B1L@SpAcDex‐ATMO‐21 NP‐treated cells. As expected, the cellular uptake of fabricated NPs showed dose‐dependent behavior. The delivery efficiency of B1L@SpAcDex‐ATMO‐21 NP‐treated cells was approximately twofold that of the group treated with NPs without B1L modification, as determined by measuring the mean fluorescence intensity of the treated cells (Figure 3c,d). In addition, the expression of miRNA‐21 in U87MG cells treated with ATMO‐21 was quantified by real‐time reverse transcription‐polymerase chain reaction (RT‐PCR) to confirm the effects of ATMO‐21 delivery (Figure S15, Supporting Information).

Recent research demonstrates that efficient endosomal escape of genes is a determinant of success or failure. Endosomal escape may be achieved via the “proton sponge” effect of the amine moieties and increased endosomal osmotic pressure by degradation of the SpAcDex material.^[^ ^41^, ^42^ ^]^ Thus, the endosomal escape of NPs in U87MG cells was carefully studied to elucidate the underlying mechanism. The colocalization ratio between endosomes and ATMO‐21 was analyzed by CLSM, which was used as a negative control of the endosomal escape efficacy of B1L@SpAcDex‐ATMO‐21 NPs. From Figure 3f, the NPs were delivered into cells and then taken up by lysosomes during 0−4 h of endocytosis. After that, the NPs gradually escaped from the lysosome. Similarly, we can observe the lysosomal escape ability of B1L@SpAcDex‐ATMO‐21 NPs in C6 cells (Figure S16, Supporting Information). The proposed mechanism for facilitating NP escape from lysosomes was that the positive charge on the surface of NP was decreased. However, the structure of B1L peptides includes many primary amine and secondary amine groups, which promotes the protonation of NPs to form a “proton sponge” and provides ATMO‐21 escape from lysosomes.

### 2.4 The Mechanism Underlying NPs Inhibition of Angiogenesis

**Figure 4:**
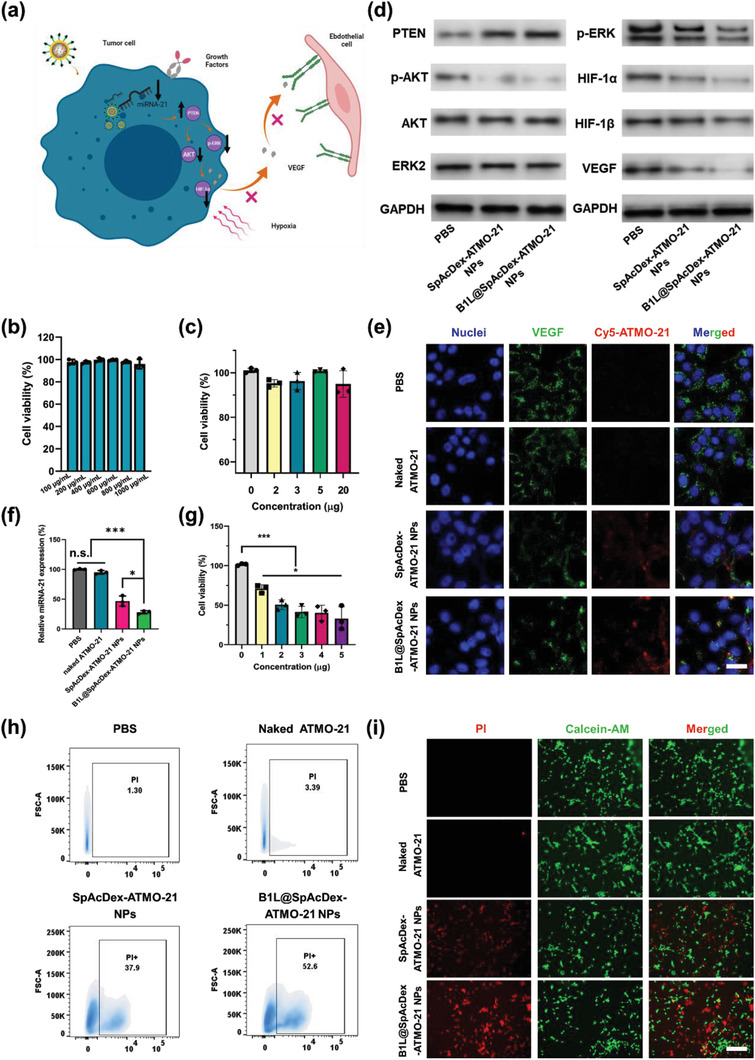
Evaluation of the capability of B1L@SpAcDex‐ATMO‐21 NPs to inhibit angiogenesis‐related genes and NP‐induced cell apoptosis. a) Proposed mechanism of as‐fabricated NPs for inhibiting the growth of tumor blood vessels. Modified icons from BioRender.com. b) Biocompatibility evaluation of B1L@SpAcDex‐ATMO‐21 NPs without ATMO‐21 when incubated with U87MG cells. Data are presented as means ± SD (*n* = 3). Or c) with ATMO‐21 when incubated with human astrocyte cells. Data are presented as means ± SD (*n* = 3). d) Relative protein expression of PTEN, p‐AKT, p‐ERK, HIF‐1*α*, and VEGF in U87MG cells in different treatment groups (phosphate buffered saline (PBS), naked ATMO‐21, SpAcDex‐ATMO‐21 NPs, B1L@SpAcDex‐ATMO‐21 NPs) evaluated by Western blotting (*n* = 3). e) Relative VEGF expression levels in different treatment groups in vitro via CLSM imaging. Scale bar, 10 µm. f) Relative miRNA‐21 expression levels in different treatment groups in vitro. Data are presented as means ± SD (*n* = 3, one‐way ANOVA, *p < 0.05, ***p < 0.001, n.s., nonsignificant). g) Cell viability of U87MG cells treated with as‐fabricated NPs at different concentrations. Data are presented as means ± SD (*n* = 3, one‐way ANOVA, *p < 0.05, ***p < 0.001). h) Flow cytometry evaluation of PI cell apoptosis. i) Live/dead cell imaging via CLSM. Scale bar, 100 µm.

After carefully investigating the efficient delivery of ATMO‐21 into tumor cells, the gene inhibition efficacy and related gene expression for inhibiting tumor blood vessel growth were studied in depth. The proposed mechanism is shown in **Figure** **4**a. First, NPs with or without ATMO‐21 were incubated with U87MG cells, C6 cells, and human astrocytes to validate their biosafety for gene delivery via MTT assay (Figure 4b,c, Figure S17a, Supporting Information) and CLSM (Figure S18, Supporting Information), which indicated that NPs showed excellent biocompatibility based on cell viability when incubated even at high concentrations (1000 µg mL^−1^) with U87MG cells and C6 cells.

Recent research has confirmed that miRNA‐21 is associated with cell proliferation and tumor blood vessel growth (Figure 4a). For this purpose, the potential antiglioma efficacy of ATMO‐21 and its potential role in influencing GBM tumor angiogenesis were investigated in vitro. U87MG cells were incubated with the as‐fabricated NPs for 48 h, and the genes regulating the tumor angiogenesis pathway were determined by Western blotting (WB) (Figure 4d, Figure S19, Supporting Information), which showed that both SpAcDex‐ATMO‐21 NPs and B1L@SpAcDex‐ATMO‐21 NPs upregulate PTEN protein expression. The difference in PTEN expression between these two groups is due mainly to the increased uptake rate of B1L‐modified NPs by U87MG cells, which further increases the expression of PTEN protein. The enhanced expression of the PTEN inhibited tumor angiogenesis by partially inactivating protein kinase B (AKT) and extracellular regulated kinase ½ (ERK½) and decreasing the expression of HIF‐1*α* to decrease VEGF expression and then inhibit tumor angiogenesis in GBM treatment. Decreased VEGF expression was also visualized by CLSM, which was consistent with the above WB results (Figure 4e). Then, real‐time RT‐PCR experiments were performed to assess miRNA‐21 expression at the miRNA level. As shown in Figure 4f, the expression trend of miRNA‐21 in the cell was PBS (100%) > naked ATMO‐21 (95%) > SpAcDex‐ATMO‐21 NPs (45%) > B1L@SpAcDex‐ATMO‐21 NPs (only 30%), suggesting that B1L‐functionalized NPs possessed excellent cell targeting ability and high miRNA‐21 inhibition efficiency. To further study the therapeutic effect of ATMO‐21‐encapsulated NPs on brain tumors cells, the proliferation of U87MG cells and C6 cells after incubation was evaluated via methyl thiazolyl tetrazolium (MTT) assays (Figure 4g, Figure S17b, Supporting Information).

We noticed that compared with other ATMO‐21‐encapsulated NPs, B1L@SpAcDex‐ATMO‐21 NPs could effectively inhibit the growth of U87MG cells (Figure S20, Supporting Information), and their cell viability decreased significantly in a dose‐dependent manner (Figure 4g), indicating a cancer cell‐penetrating NPs for highly efficient, targeted miRNA‐21 delivery with significant therapeutic efficacy. The cell apoptosis assay also validated the potent efficacy of B1L@SpAcDex‐ATMO‐21 NPs via flow cytometry based on propidium iodide (PI) staining. As displayed in Figure 4h, groups treated with PBS and naked ATMO‐21 NPs had 1.3% (panel 1) and 3.39% (panel 2) cell apoptosis, respectively. The apoptotic cell population of U87MG cells treated with B1L@SpAcDex‐ATMO‐21 NPs was 52.6% (panel 4). This value was slightly higher than that of the SpAcDex‐ATMO‐21 NPs‐treated cell group (37.9%) (panel 3), indicating that B1L‐modified NPs are potential ATMO‐21 therapeutic NPs. In addition, U87MG cells and C6 cells were stained with calcein‐AM and PI for fluorescence imaging to validate cell apoptosis. As shown in Figure 4i and Figure S17c, Supporting Information, B1L@SpAcDex‐ATMO‐21 NP‐treated cells showed a higher red fluorescence signal than the other three groups, clearly indicating the death of more cells.

### 2.5 In Vitro BBB and BTB Model

**Figure 5:**
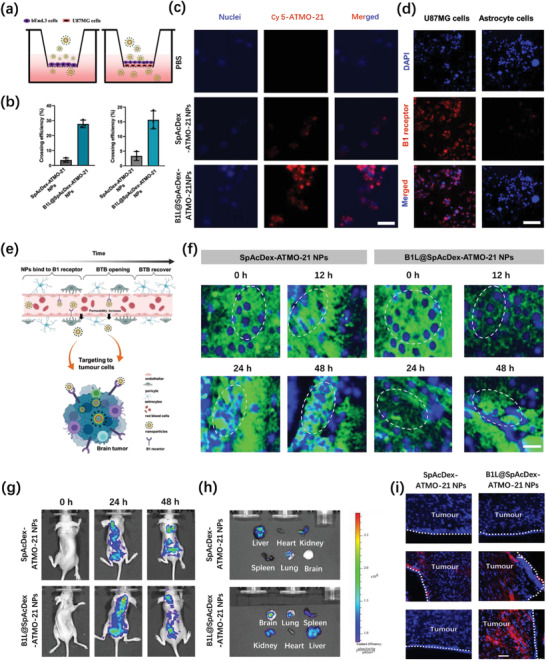
In vitro and in vivo evaluation of B1L‐mediated NP transport across the BBB and BTB. a) In vitro BBB and BTB model. b) The crossing efficiency of NPs (SpAcDex‐ATMO‐21 NPs and B1L@SpAcDex‐ATMO‐21 NPs) toward the BBB and BTB. Data are presented as means ± SD (*n* = 3). c) U87MG cell uptake within the basolateral chamber measured by CLSM. Scale bar, 20 µm. d) The expression of the B1 receptor (red fluorescence) on U87MG cells and human astrocytes visualized by CLSM. Scale bar, 20 µm. e) Proposed mechanism of B1L (agonist)‐mediated as‐fabricated NPs crossing the BBB and BTB. Modified icons from BioRender.com. f) The expression of endothelial cell (EC)‐associated tight junction protein ZO‐1 (green fluorescence) in tumor sections after treatment with SpAcDex‐ATMO‐21 NPs and B1L@SpAcDex‐ATMO‐21 NPs and measured via CLSM at different time points (0, 12, 24, and 48 h). Scale bar, 20 µm. g) Real‐time fluorescence imaging of brain tumor‐bearing mice after intravenous injection with SpAcDex‐ATMO‐21 NPs and B1L@SpAcDex‐ATMO‐21 NPs. During the in vivo fluorescence imaging, 3 mice per group were used as imaging objects. The fluorescence intensity at the mouse brain site was recorded using a small animal imager machine at 0, 24, and 48 h postinjection of as‐fabricated NPs. h) CLSM images (ex vivo) of major organs and brain tumors at 48 h postinjection. The heart, liver, spleen, lung, kidney, and brain were excised from each group of three mice after the fluorescence imaging. The fluorescence intensity at each organ was recorded separately. i) The accumulation of SpAcDex‐ATMO‐21 NPs and B1L@SpAcDex‐ATMO‐21 NPs in the brain tumor site 48 h postinjection of SpAcDex‐ATMO‐21 NPs and B1L@SpAcDex‐ATMO‐21 NPs. Scale bar, 20 µm.

It is well known that the permeability of the BBB and BTB to NPs is the greatest challenge for gene delivery, and therefore the in vitro BBB model (bEnd.3 cell culture model) and BTB model (bEnd.3 cell/U87MG cell coculture model) were built to evaluate the permeability of NPs toward the BBB and BTB. As shown in **Figure** **5**a, SpAcDex‐ATMO‐21 NPs and B1L@SpAcDex‐ATMO‐21 NPs were added to the apical cultivation chamber. The fluorescence intensity (Figure 5b) from the basolateral chamber represented the transport efficiency from these two groups after incubation with cells for 24 h. There was a noticeable difference in the fluorescence intensity in the BBB model between these two groups, which proved the importance of the B1L agonist for crossing the BBB. Similar to the BTB model, B1L@SpAcDex‐ATMO‐21 NPs showed a significantly greater ability to target tumors cells compared with SpAcDex‐ATMO‐21 NPs. CLSM imaging showed that B1L enables NPs to better target U87MG cells (Figure 5c).

### 2.6 NPs Temporarily Upregulation of BTB Permeability In Vitro

We built a transendothelial cell electrical resistance (TEER) model to investigate the capability of B1L@SpAcDex‐ATMO‐21 NPs to regulate BTB permeability temporarily in vitro. We prepared an in vitro BTB model by coculturing endothelial bEnd.3 In a transwell microplate, U87MG cells, and bEnd.3 cells are grown on the luminal and abluminal sides of the filter insert, respectively. A decreased TEER value is due to the increased barrier permeability of the cell layer and intercellular space.^[^ ^43^ ^]^ Treating the coculture cells with B1L@SpAcDex‐ATMO‐21 NPs results in a significant reduction in TEER values, with the lowest observed at 2 h post‐treatment (Figure S21, Supporting Information). Notably, the TEER values recovered 12 h after B1L@SpAcDex‐ATMO‐21 NPs treatment, suggesting the modulation of the B1L@SpAcDex‐ATMO‐21 to BTB permeability is short‐lived.

### 2.7 The Mechanism Underlying NPs‐Mediated Crossing of the BTB and Targeted Delivery of NPs In Vivo

In general, hemolysis assays are typically used to assess the safety of gene‐ or drug‐loaded NPs in an animal model.^[^ ^44^ ^]^ As shown in Figure S22, Supporting Information, B1L@SpAcDex‐ATMO‐21 NPs exhibited negligible hemolytic toxicity to red blood cells at different concentrations of NPs, demonstrating the excellent blood compatibility and stability of the ATMO‐21‐loaded NPs and their potential application as a gene delivery platform for cancer therapy. First, the expression of the B1 receptor on U87MG cells C6 cells, mouse astrocytes, and human astrocytes was measured by CLSM (Figure 5d, Figure S23, Supporting Information). As we described above, the proposed mechanism is shown in Figure 5e. B1L mediated NPs crossed the BTB by activating the B1 receptor, and then the temporarily open BTB was studied in depth. Enhanced vascular leakiness in the BTB structure is often related to a loss of tight junction proteins, which are essential for endothelial cell (EC) integrity.^[^ ^45^, ^46^ ^]^ Therefore, we quantified the EC‐associated tight junction protein ZO‐1 in tumor sections of mice treated with SpAcDex‐ATMO‐21 NPs or B1L@SpAcDex‐ATMO‐21 NPs. Consequently, we observed a significant reduction in ZO‐1 protein at 24 h postinjection (Figure 5f). Notably, the reduction in tight junction proteins is not necessarily permanent. A permanent reduction would cause serious side effects for the brain. We then noticed that the fluorescence intensity of ZO‐1 was gradually increasing at 48 h postinjection. Additionally, the tumor vascular microstructure observed by BioTEM possessed a significant porosity in the first 24 h postinjection and recovered soon after 48 h of treatment with B1L@SpAcDex‐ATMO‐21 NPs (Figure S24, Supporting Information), confirming a safe BTB opening method.

For the in vivo biodistribution of the as‐fabricated NPs, orthotopic U87MG glioma‐bearing mice were constructed as animal models. Cy5‐ATMO‐21‐loaded SpAcDex NPs (with B1L modification or not) were injected. As shown in Figure 5g, the fluorescence intensity from the tumor site was obviously enhanced at 24 h postinjection with B1L@SpAcDex‐ATMO‐21 NPs and was gradually clearing from the tumor site at 48 h postinjection, whereas there was no apparent fluorescence centered in the brain tumor for the SpAcDex‐ATMO‐21 NP‐treated group 48 h postinjection. The major organs (heart, liver, spleen, lung, kidney, and brain) were then excised from mice to analyze the distribution of NPs at 48 h via ex vivo fluorescence imaging (Figure 5h). We noticed that in the B1L@SpAcDex‐ATMO‐21 NPs‐treated group, a weak fluorescence signal existed in the brain slices, while we could not observe fluorescence in the brain sections from the SpAcDex‐ATMO‐21 NP‐treated groups (Figure S25, Supporting Information). Brain tumor tissues were obtained, and their histopathological sections were imaged by CLSM at 24 h postinjection, as shown in Figure 5i. We observed that B1L@SpAcDex‐ATMO‐21 NPs showed significant accumulation in the tumor site compared to SpAcDex‐ATMO‐21 NPs, which is in line with the above in vivo results. Based on the above experimental results, B1L‐mediated NPs were verified to cross the BTB.

### 2.8 In Vivo Tumor Inhibition and Antiangiogenesis Evaluation

**Figure 6:**
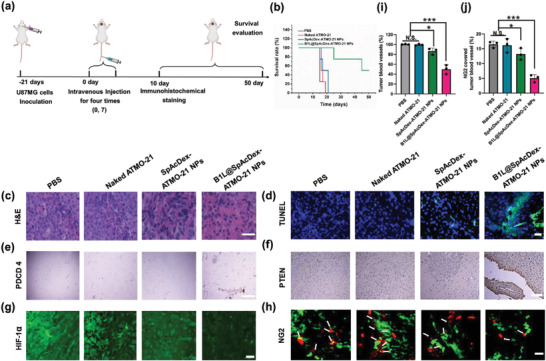
a) Schematic illustration displaying the timeline of the in vivo antitumor and antiangiogenic study. Modified icons from BioRender.com. b) Mouse survival rates when incubated with PBS, naked ATMO‐21, SpAcDex‐ATMO‐21 NPs, and B1L@SpAcDex‐ATMO‐21 NPs. (*n* = 8, Kaplan–Meier survival analysis to prepare survival plots. Statistical analysis was performed by Mantel‐Cox log‐rank test). c,d) Brain tumor tissues excised from orthotopic U87MG GBM tumor‐bearing nude mice following treatment, stained with H&E or with TUNEL for evaluating apoptosis. Scale bar, 20 µm. e) Immunofluorescence staining for evaluating PDCD 4. Scale bar, 20 µm. f) Immunofluorescence staining for evaluating PTEN. Scale bar, 20 µm. g) Representative immunofluorescence staining images of HIF‐1*α* (green fluorescence) from orthotopic U87MG GBM tumor slices. Scale bar, 50 µm. h) Representative immunofluorescence staining images of PBS (control)‐, naked ATMO‐21‐, SpAcDex‐ATMO‐21 NP‐ and B1L@SpAcDex‐ATMO‐21 NP‐treated orthotopic U87MG GBM tumor sections stained for the tumor blood vessel marker CD 31 (green fluorescence) and the pericyte marker NG2 (red fluorescence). Scale bar, 50 µm. i) Histomorphometric quantification of microvessel density in PBS (control group)‐, naked ATMO‐21‐, SpAcDex‐ATMO‐21 NP‐, and B1L@SpAcDex‐ATMO‐21 NP‐treated U87MG tumors. Data are presented as mean ± SD (*n* = 3, one‐way ANOVA, *p < 0.05, ***p < 0.001, n.s., nonsignificant). j) Quantification of pericyte‐covered brain tumor microvessel cover percentage uptake of the total vessels from the overall sectional area. Data are presented as mean ± SD (*n* = 3, one‐way ANOVA, *p < 0.05, ***p < 0.001, n.s., nonsignificant).

Based on the considerable BTB permeability of B1L@SpAcDex‐ATMO‐21 NPs in vivo, we explored their therapeutic efficacy in a glioma‐bearing mouse model. Mice were divided into four groups: group 1, PBS; group 2, naked ATMO‐21; group 3: SpAcDex‐ATMO‐21 NPs; and B1L@SpAcDex‐ATMO‐21 NPs. The detailed timeline of treatment for the mice is shown in **Figure** **6**a. As shown in Figure 6b, dextran‐based NPs encapsulating ATMO‐21 suppressed tumor growth to some extent compared with PBS (control group). Additionally, negligible tumor inhibition could be visualized in tumor slices treated with naked ATMO‐21. The B1L@SpAcDex‐ATMO‐21 NPs showed a significant inhibitory effect compared with nonmodified SpAcDex‐ATMO‐21 NPs, primarily owing to the active targeting ability of the B1L‐modified NPs. The body weights of mice showed no significant changes after treatment with B1L@SpAcDex‐ATMO‐21 NPs. However, the control groups treated with PBS and naked ATMO‐21 underwent clear bodyweight loss because of the rapid proliferation and invasion of the GBM, culminating in brain malfunctions (Figure S26, Supporting Information). The mouse survival rate indicated that B1L@SpAcDex‐ATMO‐21 NPs showed remarkable efficacy in inhibiting brain tumor proliferation and increased survival rates compared to treatment with PBS, naked ATMO‐21, and SpAcDex‐ATMO‐21 NPs.

Haematoxylin and eosin (H&E) (Figure 6c, Figure S27, Supporting Information) and terminal deoxynucleotidyl transferase dUTP nick‐end labeling (TUNEL) staining experiments were performed (Figure 6c,d). Excellent miRNA‐21 targeted inhibition efficacy was achieved in B1L@SpAcDex‐ATMO‐21 NPs‐treated mice, meaning that the B1L@SpAcDex‐ATMO‐21 NPs exhibited the highest level of tumor cell apoptosis and the lowest level of tumor cell proliferation. Furthermore, the gene expression levels of PDCD4 and PTEN in tumor tissues were investigated after treatment. Since these proteins typically influence cell apoptosis and angiogenesis in GBM, the therapeutic efficacy of ATMO‐21 will be achieved through regulating PDCD 4 and PTEN. Figure 6e shows that the mice treated with B1L@SpAcDex‐ATMO‐21 NPs showed significantly higher PDCD 4 levels than the other NPs without B1L modification, while the naked ATMO‐21 group showed similar expression levels to the PBS‐treated (control group) and SpAcDex‐ATMO‐21 NP‐treated groups. PTEN expression showed a trend similar to that of PDCD 4 (Figure 6f). Remarkably, high expression of PTEN was observed in tumor slides taken from B1L@SpAcDex‐ATMO‐21 NP‐treated mice. Therefore, highly expressed PTEN‐induced antiangiogenic therapy for GBM was evaluated by immunofluorescence staining for CD 31 (blood vessel marker), HIF‐1*α* (Figure 6g), and NG2 in tumor sections after treatment, which confirmed that B1L@SpAcDex‐ATMO‐21 NPs inhibited HIF1*α* expression compared with the control group, naked AMO‐21 and NPs without B1L functionalization.

The coverage of blood vessels with pericytes represents a vital attribute of their maturity and functionality. The quantification of NG2 pericytes showed a significant reduction in pericyte‐covered vessels upon B1L@SpAcDex‐ATMO‐21 NPs treatment (Figure 6h, white arrow marked). In contrast, the other three groups still showed extensive pericyte coverage adjacent to the tumor blood vessel, demonstrating antiangiogenic therapy by targeted delivering ATMO‐21 for GBM treatment. They are integrated with the pooled results, indicating that B1L@SpAcDex‐ATMO‐21 NPs can work effectively overcome the BTB barrier to target tumor cells, achieving continuous release of ATMO‐21 both in vitro and in vivo. All these superior properties endow these NPs with optimal antiglioma efficacy in vivo. Finally, after treatment, H&E staining of major organs was conducted to evaluate the biocompatibility of B1L@SpAcDex‐ATMO‐21 NPs, and the results further indicated that there was no evidence of in vivo toxicity (Figure S28, Supporting Information). Notably, these smart nanoplatforms can also potentially deliver and precisely release other therapeutic cargo, such as messenger RNA, DNA, enzyme, and protein, making them suitable for the treatment of various diseases.

## 3 Conclusions

In this work, ATMO‐21‐encapsulated NPs (B1L@SpAcDex‐ATMO‐21 NPs) were successfully synthesized by the double emulsion method. The whole fabrication process was reliable, repeatable, and simple, providing a stable strategy to prepare gene delivery vectors. As expected, SpAcDex NPs as the gene delivery platform achieve a high ATMO‐21 loading efficiency (over 90%) and high delivery efficiency compared with traditional vectors, including liposomes and PEI. The lysosomal escape rate is an essential index for gene delivery in cancer treatment. The as‐fabricated NPs possess an excellent lysosomal escape rate in U87MG cells, C6 cells, and human astrocytes based on the pH‐responsive “Big Bang” mechanism.

Using B1L to functionalize the surface of SpAcDex NPs enables NPs to efficiently and intelligently cross the BTB for brain tumor targeting, which shows satisfactory antitumor efficacy in mice bearing orthotopic human GBM. Most importantly, the antiangiogenic efficacy of ATMO‐21 toward orthotopic human GBM was first investigated carefully, indicating that B1L@SpAcDex‐ATMO‐21 NPs could significantly inhibit the expression of tumor blood vessel‐related genes (HIF1*α* and VEGF) by upregulating PTEN expression. This novel NPs‐mediated and multifunctional tumor microenvironment‐responsive delivery system provides a powerful and versatile platform for targeted GBM gene therapy.

## Conflict of Interest

The authors declare no conflict of interest.

## Supporting information

Supporting Information

## Data Availability

Research data are not shared.
