# Supplementary material for: Anti‐MicroRNA‐21 Oligonucleotide Loaded Spermine‐Modified Acetalated Dextran Nanoparticles for B1 Receptor‐Targeted Gene Therapy and Antiangiogenesis Therapy
Source: Adv Sci (Weinh). 2021 Dec 22;9(5):2103812. doi: 10.1002/advs.202103812 (PMC8844571; doi:10.1002/advs.202103812)
Supplement: Supplementary file 1 — Supporting Information [file ADVS-9-2103812-s001.pdf]

ADVANCED SCIENCE
Open Access

Supporting Information

for *Adv. Sci.*, DOI: 10.1002/advs.202103812

Anti-microRNA-21 Oligonucleotide loaded Spermine-Modified Acetalated Dextran Nanoparticles for B1 Receptor-targeted Gene Therapy and Antiangiogenesis Therapy

Tao Zheng, Wentao Wang, Mohsen Mohammadniaei, Jon Ashley, Ming Zhang, Ninglin Zhou,* Jian Shen,* and Yi Sun**

Supporting Information

Anti-microRNA-21 Oligonucleotide loaded Spermine - Modified Acetalated Dextran Nanoparticles for B1 Receptor-targeted Gene Therapy and Antiangiogenesis Therapy

Tao Zheng, Wentao Wang, Mohsen Mohammadniaei, Jon Ashley, Ming Zhang[a,b], Ninglin Zhou[b],* Jian Shen[b],* and Yi Sun**

1. Experimental Section

Materials

Dextran (M_w 9000-11000 g/mol) from *Leuconostoc mesenteroides*, sodium periodate, 2-methoxypropene, pyridinium p-toluenesulfonate, anhydrous dimethyl sulfoxide (DMSO), trimethylamine, spermine, sodium borohydride, methanol, 1-Ethyl-3-[3-dimethylaminopropyl]carbodiimide hydrochloride (EDC), 4’,6-diamidino-2-phenylindole (DAPI), poly(vinyl alcohol), 3-(4,5-dimethyl-2-thiazolyl)-2,5-diphenyl-2-Htetrazolium bromide (MTT), were obtained from Sigma-Aldrich Co. (St Louis, MO, USA). The ATMO-21 sequence was design according to the previous studies.[1] The ATMO-21 (sense strand: 5’-Cy5/TCAACATCAGTCTGATAAGCTA-3’), the sequences of the miR-21 primers were as follows: forward primer, 5’-AGTTGTAGTCAGACTATTCGAT-3’ and reverse primer, 5’-TCAACATCAGTCTGATAAGCTA- 3’. The sequences of the GAPDH primers were as follows: forward primer, 5’-AGACAGCCGCATCTTCTTGT-3’ and reverse primer, 5’-CTTGCCGTGGGTAGAGTCAT-3’ were obtained from Integrated DNA Technologies (Coralville). MiRNeasy Mini Kit (50) and PyroMark OneStep RT-PCR Kit (50) were bought from QIAGEN. Phosphate buffered saline (PBS) was obtained from Thermal Fisher (USA). Des-Arg^9-Kallidin was from BACHEM (Switzerland). LysoTracker™ Green DND-26 kit was obtained from Invitrogen (Carlsbad, CA), terminal deoxynucleotidyl transferase dUTP nick end labelling (TUNEL) assay kit was purchased from KeyGEN BioTECH (Nanjing, China).

Apparatus

The study of degradation behaviors of SpAcDex NPs was evaluated by a microplate reader (BioTek Synergy H1, Agilent, USA), the release profile of ATMO-21 was investigated via Nanodrop (Thermo Fisher Scientific, USA). Transmission electron microscopy (TEM) analyses of samples were carried out on a Tecnai G20 operating. Zeta potential was processed using Malvern DLS Zetasizer (United Kingdom) and Multiple–Laser ZetaView® f-NTA Nanoparticle Tracking Analyzers (German). The fluorescent imaging for cells was conducted by EVOS M5000 Imaging System (AMF5000, Thermo Fisher Scientific, USA) and Laser scanning confocal microscopy (LSM 700, Zeiss, German). The flow cytometry experiments were performed using BD FACS Calibur™, BD Biosciences Immunocytometry Systems, SanJose, CA. *In vivo* fluorescence imaging to evaluate transportability across the BBB and anti-glioma efficiency *in vivo* of NPs was observed by bioluminescence IVIS® imaging equipped with LivingImage™ software (Xenogen). H&E, TUNEL, and blood vessel staining was determined by an inverted biological microscope (Olympus BX43 microscope, Japan).

Synthesis of partial oxidation of dextran

Dextran (5.0 g, 30.9 mmol, M_w 9000-11 000 g/mol, from *Leuconostoc mesenteroides*) was dissolved in 20 mL MQ water. After adding sodium periodate (1.1 g, 51.4 mmol), the solution was stirred for 5 h at room temperature. The product was purified by dialysis of the solution against distilled water using a regenerated cellulose membrane with an MWCO of 3500 g/mol. The water was changed 5 times, and the sample was lyophilized to obtain a white powder (4.2 g).

Synthesis of acetalated dextran (AcDex) with partial oxidization

Acetalation of partially oxidized dextran was synthesized according to previous protocol with some changes.[2] Briefly, partially oxidized dextran (3.0 g) was reacted with pyridinium p-toluenesulfonate (46.8 mg) with 2-methoxypropene (10.6 mL) in anhydrous DMSO (20 mL) during 2 h under an N_2 atmosphere to obtain the partially oxidized AcDeX, yielding partially oxidized AcDex (3.5 g). The degree of functionalization was determined by 1H NMR spectroscopy in d_6-DMSO. ^{1}H NMR (400 MHz, d_6-DMSO): δ 1.33 (s, br, acetal), 3.16 (br, acetal), 3.48, 3.55-4.01, 4.90, 5.11 (br, dextran).

Synthesis of spermine-Ac-DEX (SpAcDex)

Acetalation of partially oxidized dextran was synthesized according to previous protocol with some changes.[1] Partially oxidized AcDex (2.0 g) was stirred with spermine (4.0 g) in 10 mL DMSO at 50 °C for 22 h due to the low solubility of spermine in DSMO at room temperature. The reduction was processed for 72 h at room temperature by adding sodium borohydride to the reaction solution two times successively with 1 g each time (totally 2.0 g) to the DMSO solution. Then, the final reaction solution was precipitated in MQ water (200 mL). The product was isolated by centrifugation at 5000 rpm for 30 min, and the resulting pellet was washed thoroughly with MQ water 3 times. The final product was obtained by lyophilization, yielding SpAcDex (1.75 g) as a white powder. The degree of

functionalization was determined by elemental analysis using the nitrogen content. ^{1}H NMR (400 MHz, d_6-DMSO): δ 1.40 (s, br, acetal), 1.45, 1.51, 2.55, 2.67 (br, spermine), 3.14 (br, acetal), 3.45, 3.50-4.10, 4.90, 5.10 (br, dextran).

Preparation of SpAcDex encapsulating ATMO-21 NPs (SpAcDex-ATMO-21 NPs)

SpAcDEX polymer encapsulating ATMO-21 was fabricated by applying a double emulsion (water/oil/water) way. The stock ATMO-21 solutions (1 mg/mL) were prepared in nuclease-free distilled water. SpAcDex (25 mg) was dissolved in cold dichloromethane (DCM, 600 μL) prepared in advance. The stock ATMO-21 solution (25 μL) was added to undergo a sonication for 30 s every 15 seconds on ice using a probe sonicator (Qsonica L.L.C, USA) flat tip, an output setting of 10, and a duty cycle of 80%. Then the poly(vinyl alcohol) (PVA, Mw 9000-10000 g/mol, 80% hydrolyzed) (1 mL, 3% w/w in MQ water) was added into the primary emulsion solution and further sonicated for an additional 30 s on ice using the same settings. The resulting double emulsion was immediately poured into a second PVA solution (5 mL, 0.3% w/w in MQ water) and stirred for 6 h at room temperature for evaporating the DCM solvent. The NPs were isolated by centrifugation (14800 rpm, 10 min) and washed with PBS by sonication and vortexing. The washed particles were resuspended in PBS and lyophilized further.

Preparation of empty NPs

NPs that did not package ATMO-21 were prepared similarly as above, replacing that of ATMO-21 with MQ water.

Preparation of Des-Arg^9-Kallidin (B1L) modified NPs (B1L@SpAcDex-ATMO-21 NPs)

The Des-Arg^9-Kallidin (B1L, 25 mg) was dissolved in the MQ water and firstly activated by 1-Ethyl-3-[3-dimethylaminopropyl]carbodiimide hydrochloride (EDC) (10 mg) for 30 min. Then the SpAcDex-ATMO-21 NPs (25 mg) were added to the above solution and reacted for 16 h. The NPs were obtained by centrifugation (14800 rpm, 10 min) and washed with PBS (3 times) via sonication and vortexing, then centrifugation and removing the supernatant. The NPs were then lyophilized by mixing them with 60 mg/ml sucrose as a cryoprotectant.[3]

Preparation of PEI25K@ATMO-21 NCs

To form PEI25k@ATMO-21 NCs, Cy5-ATMO-21s (25 μg) and PEI25k (25 mg, Polyethylenimine, branched) were mixed and incubated for 30 min at room temperature.

Preparation of Liposome@ATMO-21 NPs

To prepare Liposome@ATMO-21 NPs, add 1 ml of an aqueous solution containing the Cy5-ATMO-21 solute (55 μg) to be encapsulated into the vial containing cholesterol (9 μmol) L-α-Phosphatidylcholine (63 μmol from egg yolk), and stearylamine (18 μmol) at the desired temperature

(> 4 °C) and mix well by vortexing for 30 seconds. A homogenous milky suspension was generated. Then the suspension was agitated by another 30 min at room temperature to improve the encapsulation efficiency. The Liposome@ATMO-21 NPs were obtained by centrifugation (14800 rpm, 10 min) and washed with PBS (3 times) via vortexing, followed by centrifugation and removing the supernatant. The NPs were then lyophilized by mixing them with 60 mg/mL sucrose as a cryoprotectant.

TEM imaging

The isolated as-fabricated NPs were diluted in MQ water, dropping the droplets onto the surface of the copper grid. The morphology and size of the NPs were investigated at a voltage of 120 kV.

Quantification of loaded ATMO-21

Particles containing ATMO-21 were suspended at a 25 mg/mL concentration in an acetate buffer (pH 5) and incubated at 37°C under gentle agitation for 48 h. The final solution was centrifuged at high speed (14800 rpm) for 5min to remove 100 μL as test samples using the Nanodrop. The loading efficiency of ATMO-21 was obtained as follows:

$$Loading\ efficiency\ (\%) = \frac{\text{Concentration ATMO} - 21 \text{ detected in Nanodrop}}{\text{Total concentration of ATMO} - 21}$$

pH-Dependent degradation of as-fabricated NPs

The NPs without ATMO-21 were dissolved 1 mg/mL in either PBS (pH 7.4) or acetate buffer (pH 5.0) and incubated at 37 °C under gentle agitation. At different time points, 100 μL aliquots were obtained and centrifuged at 14 800 rpm for 5 min to take 20 μL supernatant. The collected supernatant samples were analyzed for the presence of reducing polysaccharides using a microplate reductometric bicinchoninic acid based assay (UV absorption at 562 nm) according to the manufacturer's protocol (BCA Protein Assay Kit).

pH-Dependent release of ATMO-21 from as-fabricated NPs

The NPs encapsulating ATMO-21 were dissolved 1 mg/mL in either PBS (pH 7.4) or acetate buffer (pH 5.0) and incubated at 37°C under gentle agitation. At various time points, 100 μL aliquots were obtained, centrifuged at 14 800 rpm for 5 min to take 20 μL supernatants. The quantity of ATMO-21 in the supernatant samples was determined by using Nanodrop.

Cell culture

U87MG cell lines (malignant glioma cells), C6 cells (mouse glioma cell line), mouse astrocytes, and human astrocytes were obtained from the American Type Culture Collection (ATCC). All cell lines were cultured in a DMEM media supplemented with 10% (v/v) fetal bovine serum (FBS), 1%

streptomycin and penicillin. Cell incubations were performed in an incubator (37 °C, 5 % CO_2). The culture medium was changed every 2 days, and then the cells were collected by treated with 0.25% trypsin-EDTA solution after reaching.

In vitro **cellular uptake**

U87MG cells, C6 cells and human astrocytes were grown in DMEM supplemented with 10% FBS. The cells were seeded at a density of 10^6 cells per well in 12-well plates and incubated for 24 h at 37 °C in a 5% CO_2 incubator. Cy5-ATMO-21 was loaded in the liposome, AcDex NPs, SpAcDex-ATMO-21 NPs, and B1L@SpAcDex-ATMO-21 NPs as described above. Each group was incubated with NPs at a fixed concentration of 0.1 μg/mL. The NPs were added to the cells and incubated for 4 h at 37°C. Then, the medium was replaced with the fresh medium and incubated for an additional 20 h. The cells were harvested, and Cy5-positive cells were analyzed by confocal laser scanning microscopy (CLSM, LSM 700, Zeiss, German) and flow cytometry (BD FACS Calibur™, BD Biosciences Immunocytometry Systems, SanJose, CA).

Lysosomal escape capability of NPs in U87MG cells and C6 cells

To explore the lysosomal escape ability of as-fabricated NPs, U87MG cells and C6 cells were incubated with B1L@SpAcDex-ATMO-21 NPs and staining by the LysoTracker™ Green DND-26 (1 mM, 0.2 μL) at different time points and DAPI at 37°C , and using CLSM imaging to localize the NPs' distribution, as well as their lysosomal escape capability.

Flow cytometry experiments

U87MG cells and human astrocytes were grown in DMEM supplemented with 10% FBS. The cells were seeded at a density of 10^6 cells per well in 12-well plates and incubated for 24 h at 37 °C in a 5% CO_2 incubator. The cells were harvested after 24 h incubation with as-fabricated NPs, and Cy5-positive cells (cellular uptake of NPs) or PI-positive cells (cell apoptosis) were analyzed flow cytometry (BD FACS Calibur™, BD Biosciences Immunocytometry Systems, SanJose, CA).

Cell cytotoxicity

MTT cell growth assay is a colourimetric assay that can test the cytotoxicity of NPs to cells. Briefly, U87MG cells, C6 cells and human astrocytes were seeded into 96-well plates at 2×10^4/well. Then the cells were incubated with different concentrations of B1L@SpAcDex-ATMO-21 NPs for 24 h after undergoing a 24 h adherent growth. The MTT solution (50 µL, 2 mg/mL) was added to each well as follows. In addition, as the control group, NPs without ATMO-21 were also incubated cells to assess their biosafety to tumour cells or normal healthy cells. The microplate reader (BioTek Synergy H1, Agilent, USA) was used to test the optical density at 570 nm.

The therapeutic efficacy of as-fabricated NPs towards tumour cells was also investigated through live/dead staining. The fresh medium containing Calcein-AM (5 μg/mL) and PI (10 μg/mL) was added into the culture well and incubated with NPs for 20 min. After that, the warm PBS solution was used to wash the cells, further visualized by the CLSM imaging to assess their antitumour efficacy.

Haemolysis evaluation

RBCs were acquired by centrifuging 1 mL whole blood with anticoagulation at 4000 rpm for 5 min. The RBCs were suspended in PBS at a volume ratio of 1:10. Afterwards, 200 μL of B1L@SpAcDex-ATMO-21 NPs solutions with different concentrations were mixed with above centrifuged RBCs. After being stationary in PBS (negative control) and MQ water (positive control) for 1 h, the supernatant was obtained after centrifuging and was further accessed to calculate the hemolysis rate. All mixtures were maintained at 37 °C for 3.5 h and then centrifuged at 4000 rpm for 5 min. The optical density (OD) at 541 nm was obtained using a microplate reader to calculate hemolysis rate, and the calculation equation was as follow:

$$\text{Haemolysis rate (\%)} = \frac{\text{OD}_{\text{treatment group}} - \text{OD}_{\text{negative}}}{\text{OD}_{\text{positive}} - \text{OD}_{\text{negative}}} \times 100\%$$

$\text{OD}_{\text{treatment group}}$, $\text{OD}_{\text{negative}}$, and $\text{OD}_{\text{positive}}$ represent the absorption values of the sample, negative group, and positive control, respectively. EVOS M5000 imaging microscope was used for observing the shape of RBCs. 200 μL of B1L@SpAcDex-ATMO-21 NPs with different concentrations were incubated with 1 mL of the attenuated RBCs suspension in tubes for 4 h. The mixtures were centrifuged to acquire RBCs. Then the droplets containing RBCs was dropped on a glass slide to visualize through microscopy imaging.

MiRNA-21 real-time RT-PCRT

The U87MG cells were prepared in micro-tubes. The running progress was conducted according to the provided protocol. The sequences of the miR-21 primers were as follows: forward primer, 5’-AGTTGTAGTCAGACTATTCGAT-3’ and reverse primer, 5’-TCAACATCAGTCTGATAAGCTA-3’. The sequences of the GAPDH primers were as follows: forward primer, 5’-AGACAGCCGCATCTTCTTGT-3’ and reverse primer, 5’-CTTGCCGTGGGTAGAGTCAT-3’.

***In vitro* BBB and BTB model**

Both *in vitro* BBB and BTB model was built based on the previous report.[4] Based on the BBB model *in vitro*, the bEnd.3 cells (1×10^5/well) were seeded into the apical chamber, which was put into the 24-well plates, and the lower chamber was filled with DMEM medium, which was replaced with fresh medium every two days. The trans-endothelial electrical resistance (TEER) was used to record the

tightness of the cell membrane until the density of the cultured cells was up to 100%. The following steps were processed when the TEER value was over 300 Ω·cm^2.

Cy5-labelled SpAcDex-ATMO-21 NPs or B1L@SpAcDex-ATMO-21 NPs were added to the apical chamber at a 1mg/mL concentration. The transwells were then put into a shaking bath (37 °C, 60 rpm) for 24 h of incubation. Then, 600 μL of aliquots were collected from the basolateral chamber and replaced with the same volume of fresh DEME medium. The transport efficiency of NPs was evaluated by determining the fluorescence intensity in collected aliquots.

The *in vitro* BTB model, a similar condition, was used based on the *in vitro* BBB model. bEnd.3 cells and U87MG cells were selected to build a BTB model to study the penetration ability of NPs and verify the targetability of B1L. In brief, U87MG cells (1 × 10^5/well) were seeded into the apical chamber, which was put into the 24-well plates. After that, the bEnd.3 cells (1 × 10^5/well) were seeded into the apical chamber and growth on the U87MG cells. The basolateral chamber was filled with DMEM medium, which was replaced with fresh medium every 2 days. The TEER was used to record the tightness of the cell membrane until the density of the cultured cells was up to 100%. Cy5-labelled SpAcDex-ATMO-21 NPs or B1L@SpAcDex-ATMO-21 NPs were injected into the apical chamber and cultured for 24 h. Furthermore, the fluorescence intensity of 600 μL of collected aliquots from the basolateral chamber was analyzed by a microplate reader. The cell uptake was observed by CLSM. The penetration ability of NPs was evaluated by determining the fluorescence intensity visualized by CLSM.

Animal model

All experiments involving animals were performed in compliance with relevant ethical regulations in adherence with the Nanjing University and Nanjing Normal University for the Care and Use of Laboratory Animals, Nanjing, China. U87MG human glioma cells were purchased from ATCC and maintained in DMEM media with 10% FBS. Six-week-old NCr nude female mice were used to generate intracranial orthotopic U87MG gliomas. Briefly, mice were anaesthetized using 2% isoflurane, and their heads were immobilized in a stereotactic headframe using atraumatic ear bars. A burr hole was made using a steel drill bit (Plastics One, Roanoke, VA, USA) 1.4 mm right of the sagittal and 1mm anterior to the lambdoid suture. Tumours were allowed to grow for 14 days before treatment. Intracranial tumour growth was monitored *in vivo* using magnetic resonance imaging.

***In vivo* fluorescence imaging**

SpAcDex-ATMO-21 NPs and B1L@SpAcDex-ATMO-21 NPs were respectively injected into orthotopic brain tumour-bearing mice. Fluorescence intensity scanning was conducted at different time points after intravenous injection (0, 24 h, and 48 h) by Maestro 2 Multispectral Small-animal

Imaging System. After treatment, the major organs (heart, liver, spleen, lung, brain, and kidney) were collected for fluorescence intensity scanning.

In vivo treatment for orthotopic tumour model

The orthotopic brain tumour-bearing mice were divided into four groups: group 1 PBS; group 2 naked ATMO-21; group 3 SpAcDex-ATMO-21 NPs; and group B1L@SpAcDex-ATMO-21 NPs, in which 3 mice were used in each group. The body weight of each mouse was recorded every 2 days in 14 days. The related antiangiogenesis gene and protein were carefully investigated by immunofluorescence staining. After treatment, the major organs (heart, liver, spleen, lung, kidney) were removed for histopathological examination (H&E staining).

Survival rate evaluation

Following administration of the PBS (control group), naked ATMO-21, the SpAcDex-ATMO-21 NPs, and the B1L@SpAcDex-ATMO-21 NPs, in which 8 mice were used in each group, every day all animals were checked regularly, the time of death and the remaining number of animals were recorded. The graphic was drawn with the number of animals against time, and the NPs formulations were evaluated in terms of survival time of the mice. The mortality data were subjected to Kaplan–Meier survival analysis to prepare survival plots. Statistical analysis was performed by Mantel-Cox log-rank test.

Statistical analysis

Unless otherwise specified, all statistical analyses were performed by one-way analysis of variance (ANOVA) using GraphPad Prism 8 (GraphPad Software Inc.). The sample size for each group was ≥ 3, where n represents biological replicates. All numerical data are reported as mean ± SD. A Kaplan–Meier survival curve was generated, and a Mantel-Cox log-rank test was applied to compare survival distributions. For all survival experiments, n represents the number of animals per group. All reported P were considered to be statistically significant at $*P < 0.05$, $**P < 0.01$, $***P < 0.001$, n.s., nonsignificant.

2. Supporting Figures

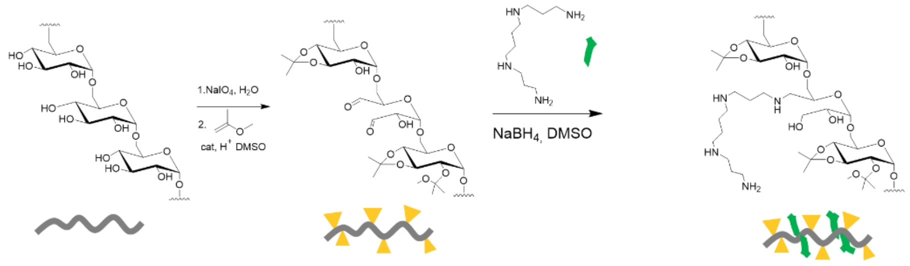

Figure S1. Synthesis route of spermine-modified Ac-DEX polymers (SpAcDex).

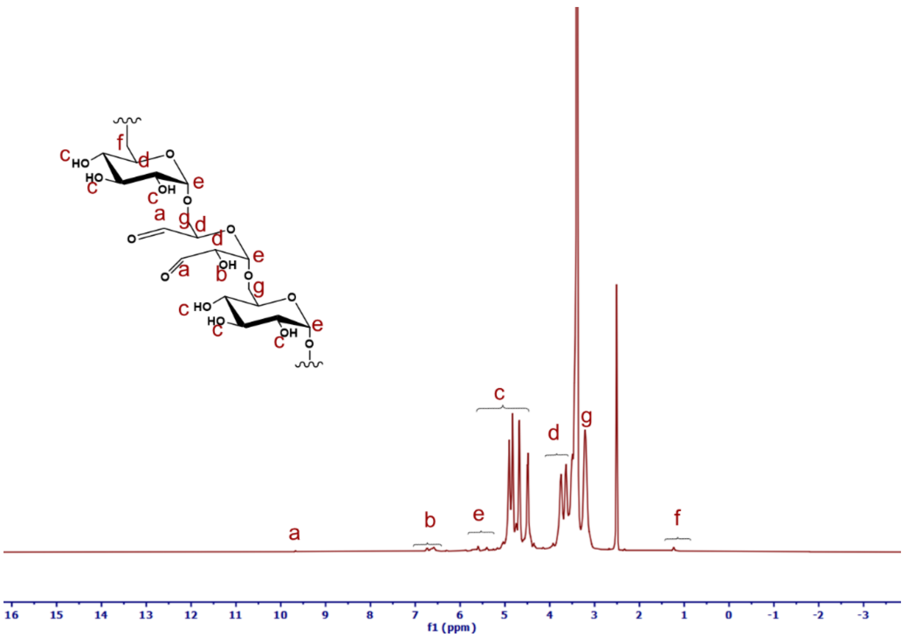

Figure S2. ^{1}H NMR spectrum of partial oxidation of dextran in d_6-DMSO.

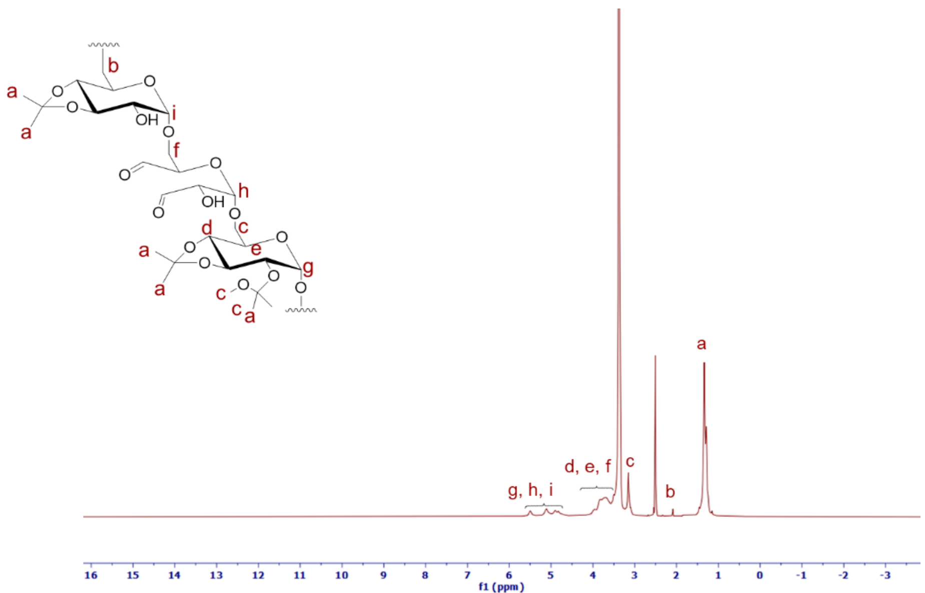

Figure S3. ^{1}H NMR spectrum of Acetalated Dextran (AcDex) with partial Oxidization in d_6-DMSO.

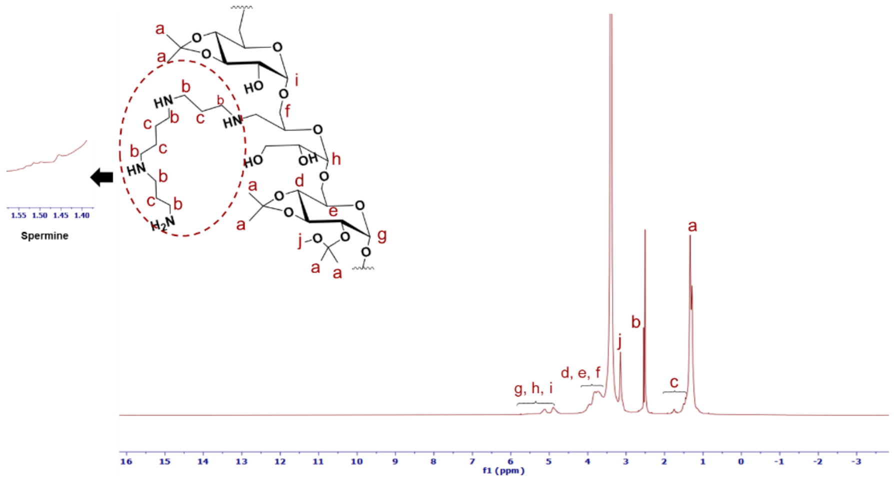

Figure S4. ^{1}H NMR spectrum of spermine-modified Ac-DEX polymers (SpAcDex) in d_6-DMSO.

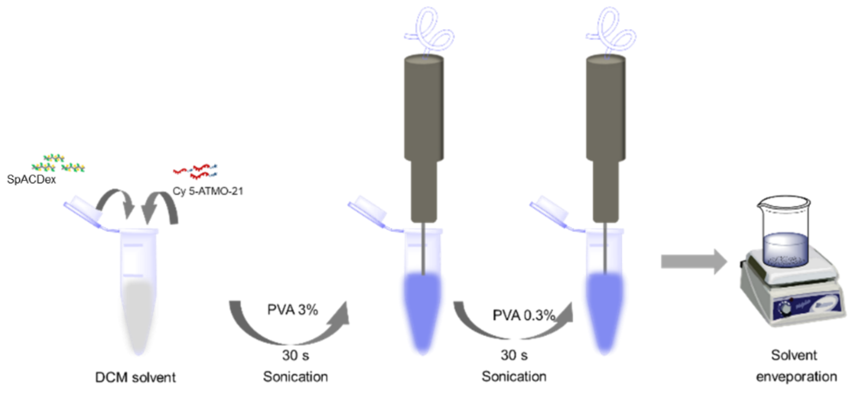

Figure S5. Preparation of SpAcDex NPs encapsulating ATMO-21 (SpAcDex-ATMO-21 NPs) using double emulsion method.

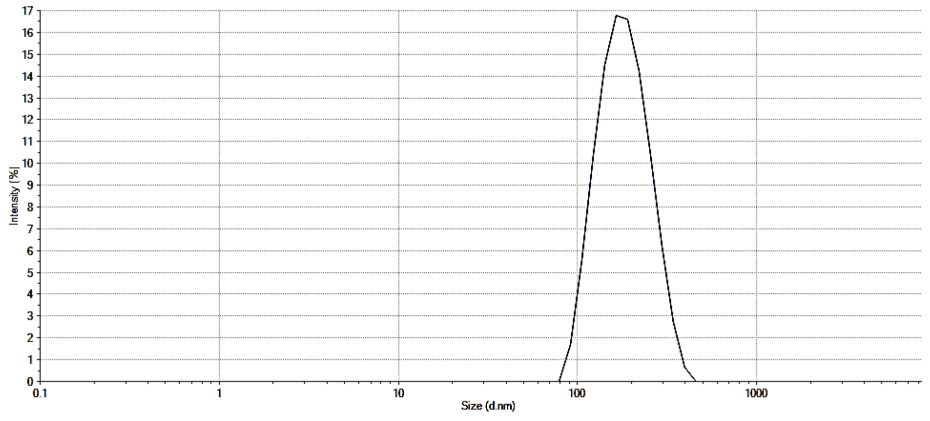

Figure S6. Size distribution of B1L@SpAcDex-ATMO-21 NPs determined by DLS.

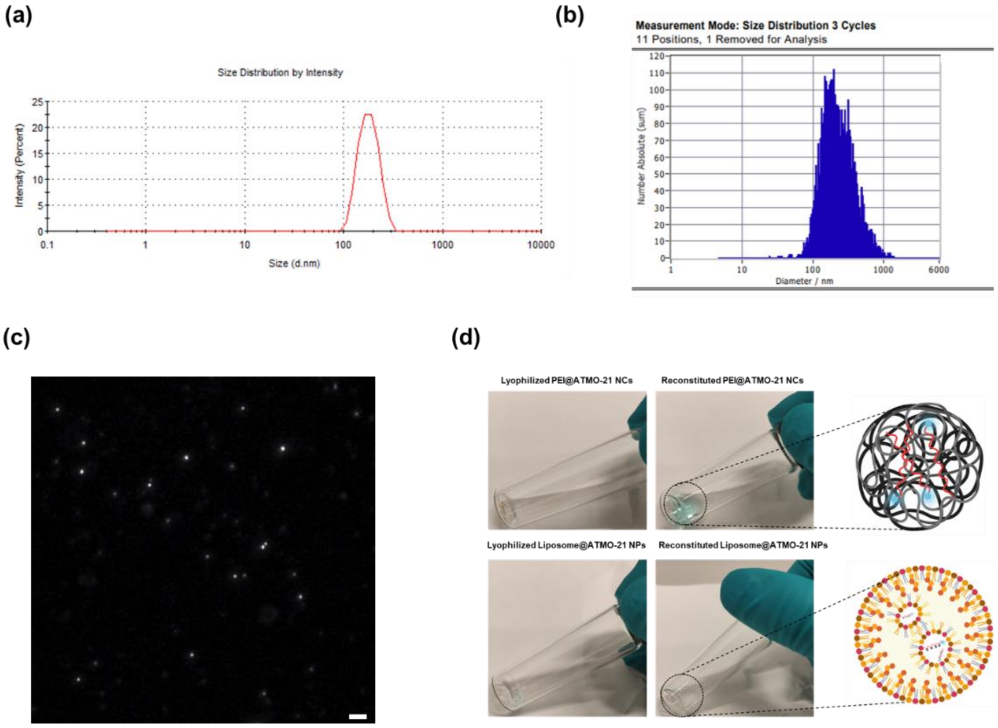

Figure S7. Size distribution of (a) PEI@ATMO-21 NCs and (b) Liposome@ATMO-21 NPs determined by DLS and Nanoparticle Tracking Analyzer (NTA), respectively. (c) Morphology imaging of Liposome@ATMO-21 NPs determined by NTA. (d) The figure of reconstituted PEI@ATMO-21 NCs and (d) Liposome@ATMO-21 NPs after the lyophilization. Scale bar, 1 μm

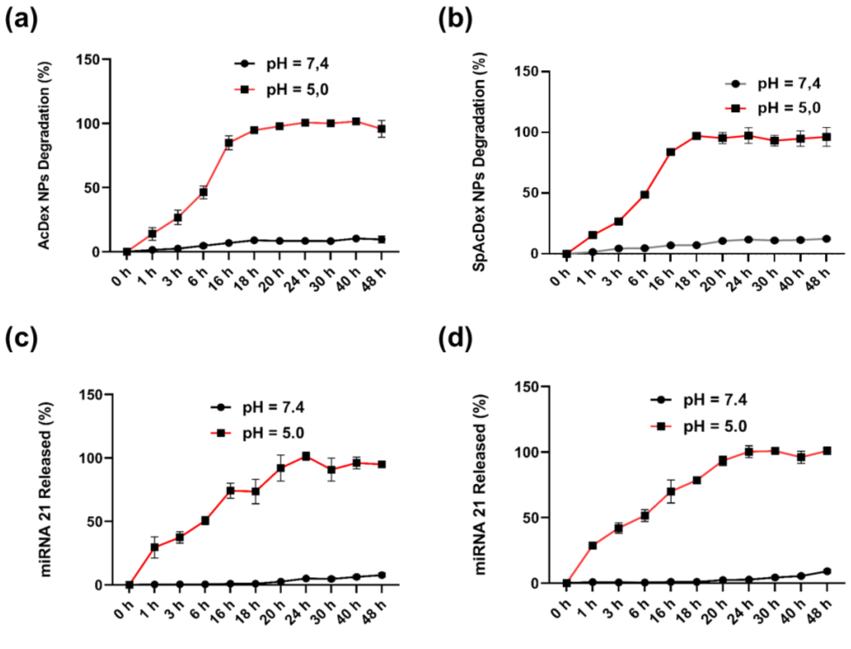

Figure S8. (a), (b) Degradation of NPs (without ATMO-21) when incubated at pH 7.4 (black square) and pH 5 (black circles), as determined by analysis of released soluble dextran. (c),(d) Release of ATMO-21

from NPs when incubated at pH 7.4 (black square) and pH 5 (black circles). as determined by analysis of released ATMO-21 in supernatant. Data are presented as means ± SD (n = 3).

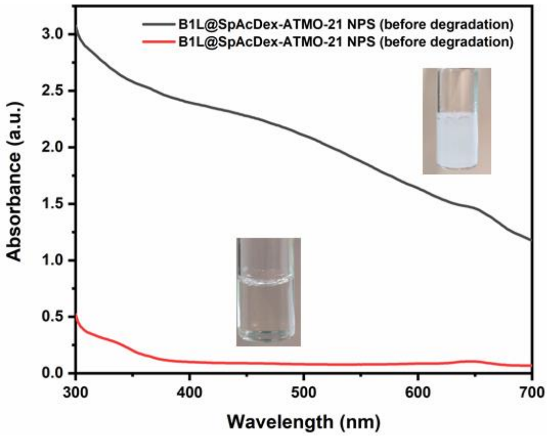

Figure S9. *In vitro* delivery of ATMO-21 with different vectors. Flow cytometry analysis with PBS, naked ATMO-21, PEI@ATMO-21 NCs, Liposome@ATMO-21 NPs, SpAcDex-ATMO-21 NPs, and B1L@SpAcDex-ATMO-21 NPs incubated U87MG cells.

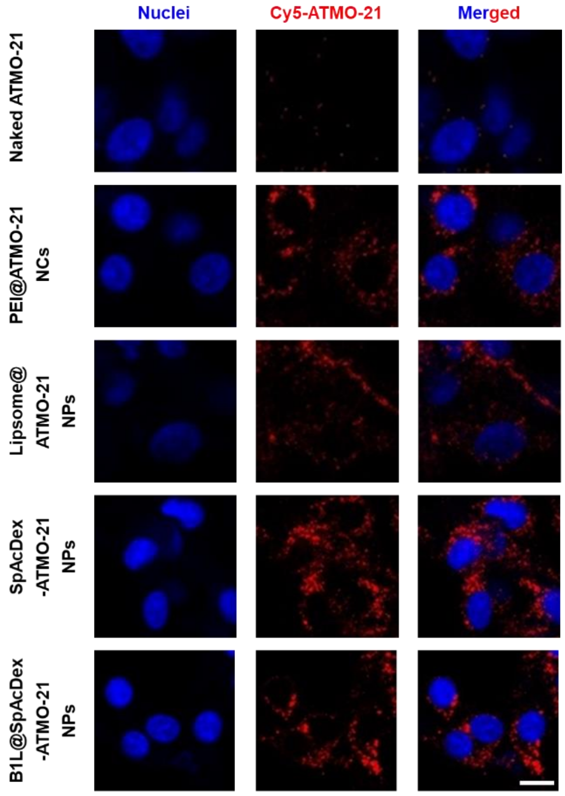

Figure S10. *In vitro* delivery of ATMO-21 with different vectors in C6 cells. CLSM analysis with the naked ATMO-21, PEI@ATMO-21 NCs, Liposome@ATMO-21 NPs, SpAcDex-ATMO-21 NPs, and B1L@SpAcDex-ATMO-21 NPs incubated C6 cells. Scale bar, 10 μm

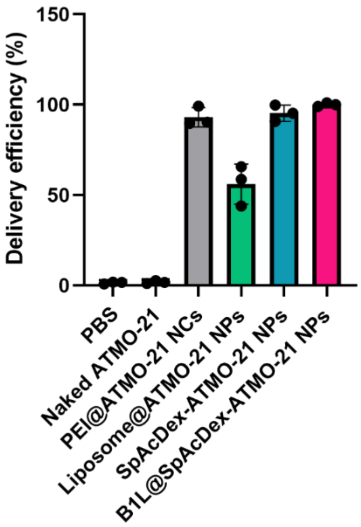

Figure S11. *In vitro* delivery of ATMO-21 with different vectors. Flow cytometry analysis with PBS, Naked ATMO-21, PEI@ATMO-21 NCs and Liposome@ATMO-21 NPs, SpAcDex-ATMO-21 NPs, and B1L@SpAcDex-ATMO-21 NPs incubated U87MG cells. Data are presented as means ± SD (n = 3).

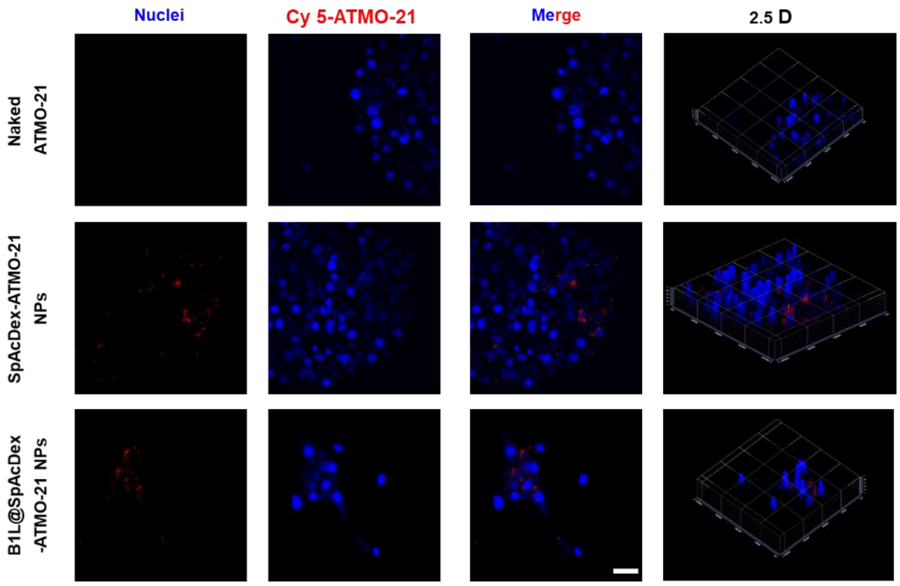

Figure S12. *In vitro* delivery of ATMO-21 with different vectors in human astrocytes. CLSM analysis with the naked ATMO-21, SpAcDex-ATMO-21 NPs, and B1L@SpAcDex-ATMO-21 NPs incubated human astrocytes. Scale bar, 20 μm

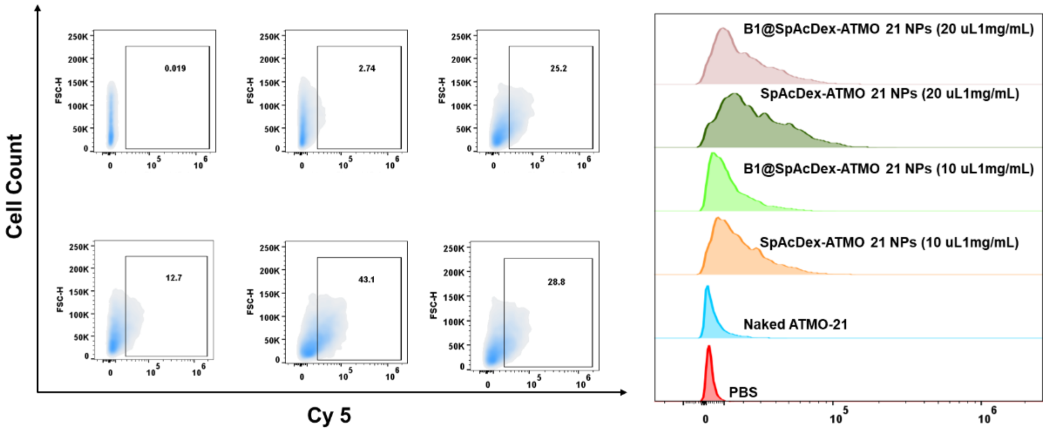

Figure S13. *In vitro* delivery of ATMO-21 with different vectors in human astrocytes. Flow cytometry analysis with the naked ATMO-21, SpAcDex-ATMO-21 NPs, and B1L@SpAcDex-ATMO-21 NPs incubated human astrocytes.

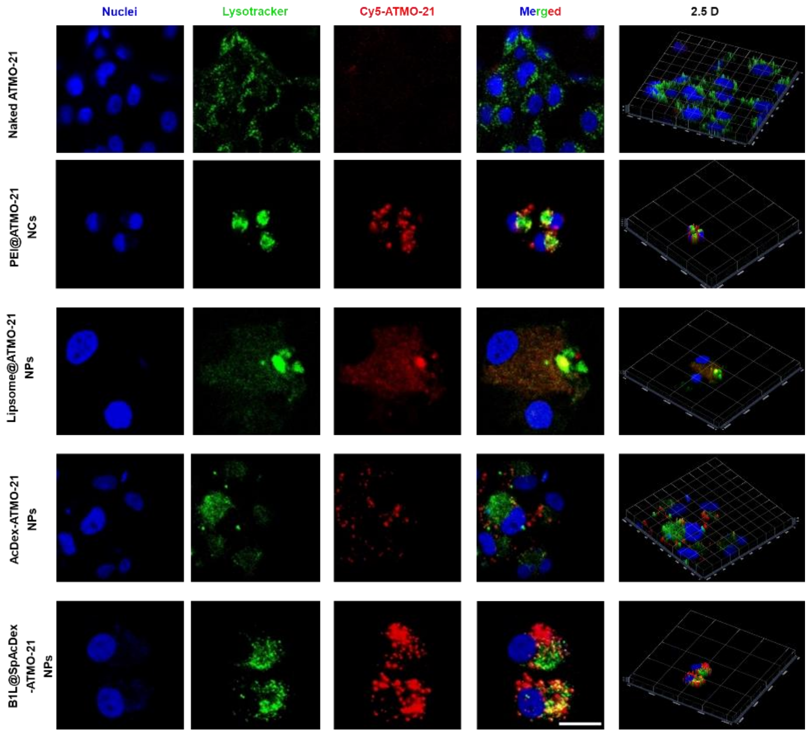

Figure S14. CLSM images of endosomal escape of as-fabricated NPs (naked ATMO-21, PEI@ATMO-21 NCs, Liposome@ATMO-21 NPs, SpAcDex-ATMO-21 NPs, and B1L@SpAcDex-ATMO-21 NPs) in C6 cells measured by CLSM. Scale bar, 10 μm

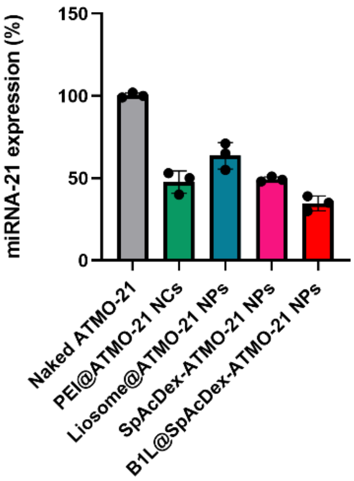

Figure S15. Relative miRNA-2l expression levels after delivery of ATMO-21 *in vitro*. Real-time RT-PCR analysis was performed for quantification of miR-21 levels in U87MG cells. Data are presented as means ± SD (n = 3).

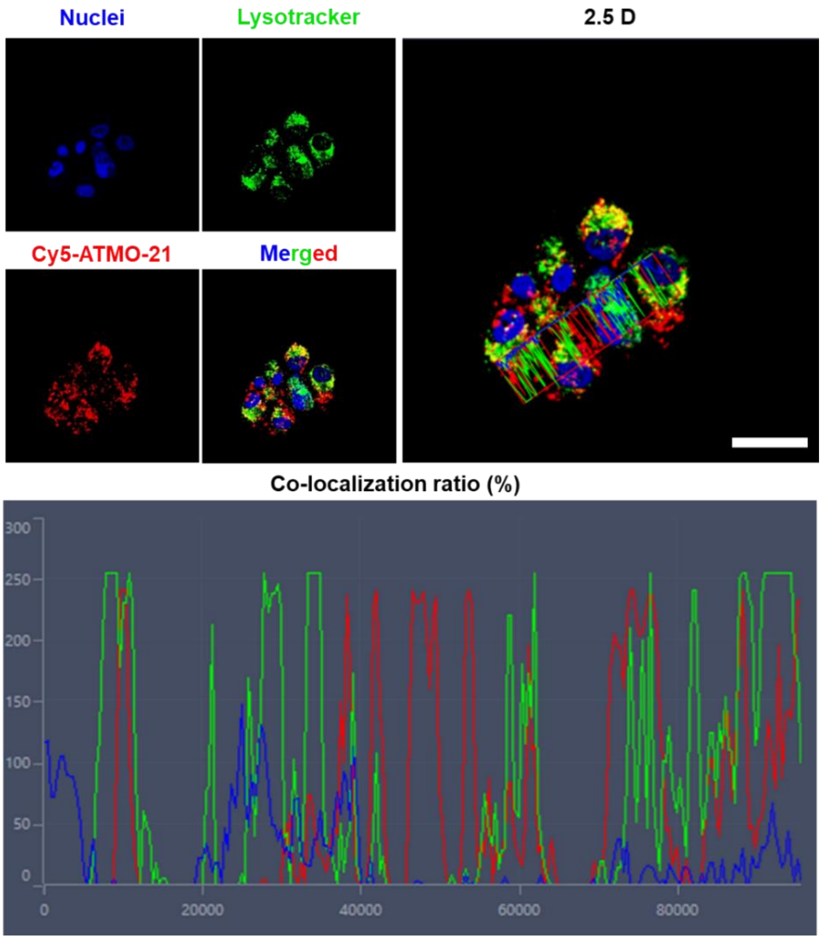

Figure S16. pH-responsive endo/lysosomal escape of B1L@SpAcDex-ATMO-21 NPs. The corresponding colocalization fluorescence intensity and the colocalization ratios between NPs (red fluorescence) and

endososomes (green fluorescence) in C6 cells when incubated with B1L@SpAcDex-ATMO-21 NPs for 10 h measured by CLSM. Scale bar, 10 µm

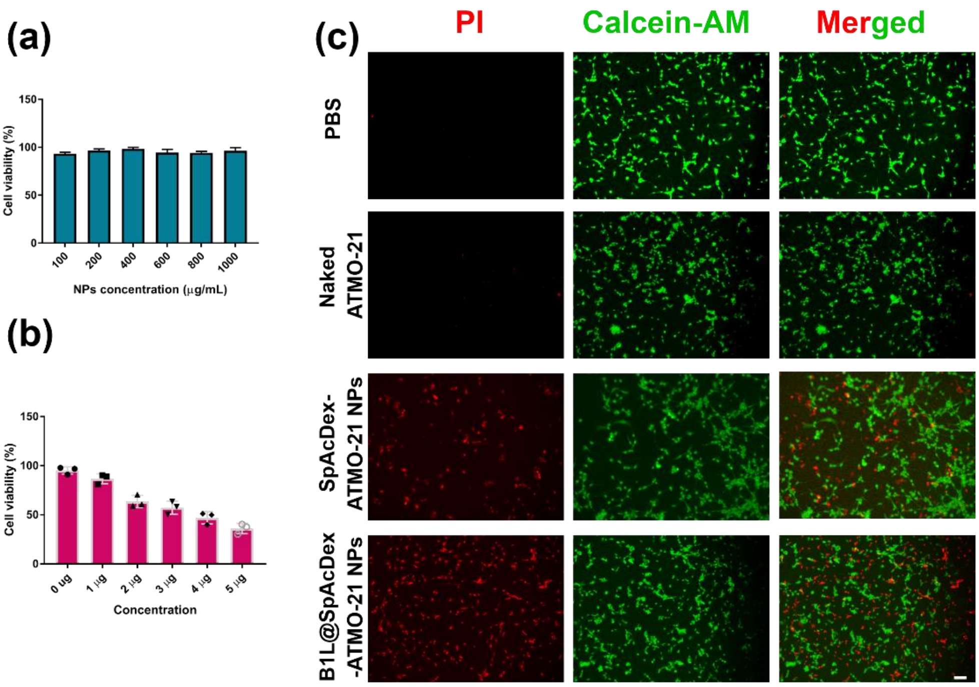

Figure S17. Biocompatibility evaluation of B1L@SpAcDex-ATMO-21 NPs **(a)** without ATMO-21 or **(b)** with ATMO-21 when incubated with C6 cells. Data are presented as means ± SD (n = 3). **(c)** Live/dead cell imaging via CLSM. Scale bar, 20 µm

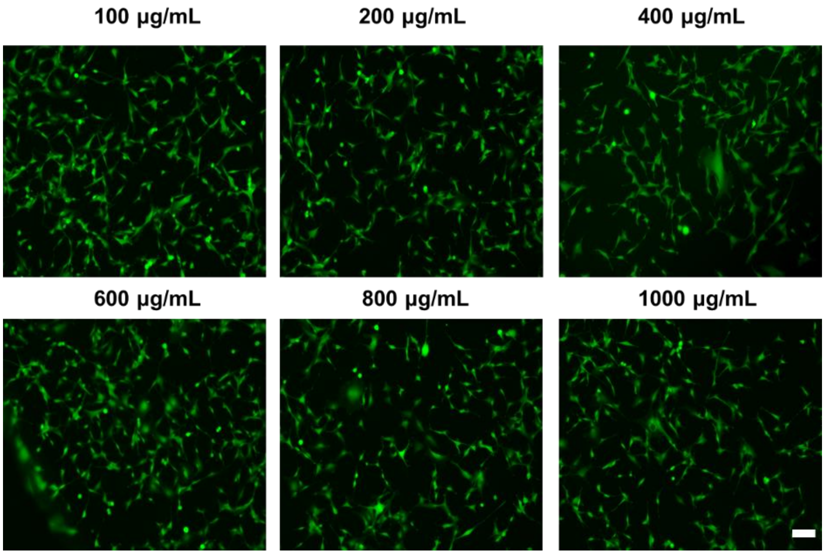

Figure S18. *In vitro* cytotoxicity evaluation of as-fabricated NPs without gene to cancer cells. Live/dead assay of U87MG cells after incubation with different concentration of B1L@SpAcDex-ATMO-21 NPs without ATMO-21 for 24 h and subsequent staining with Calcein-AM/PI probe. Scale bar, 20 µm

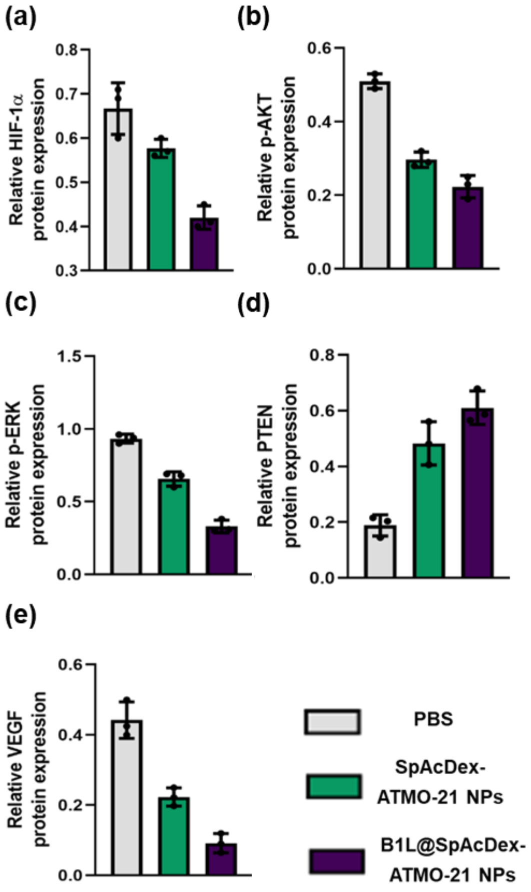

Figure S19. Relative protein expression of (a) HIF-1α, (b) p-AKT, (c) p-ERK, (d) PTEN, and (e) VEGF on U87MG cells evaluated by Western blotting. Data are presented as means ± SD (n = 3).

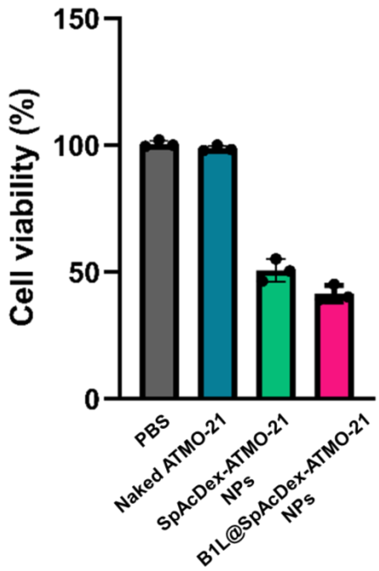

Figure S20. Cell viability of U87MG cells when incubated with as-fabricated NPs (PBS, naked ATMO-21, SpAcDex-ATMO-21 NPs, and B1L@SpAcDex-ATMO-21 NPs) and evaluated by MTT assay. Data are shown as mean ± SD (n=3).

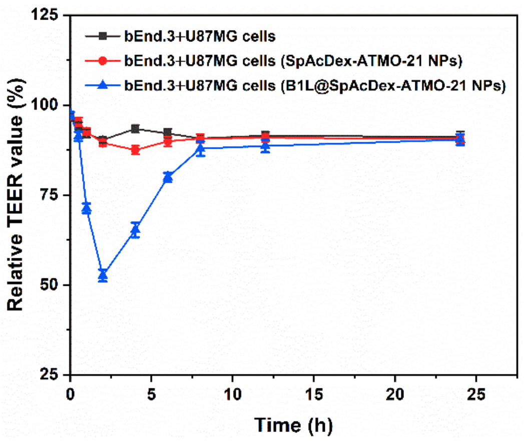

Figure S21. TEER values recorded immediately after addition of SpAcDex-ATMO-21 NPs and B1L@SpAcDex-ATMO-21 NPs. Data are shown as mean ± SD (n=3).

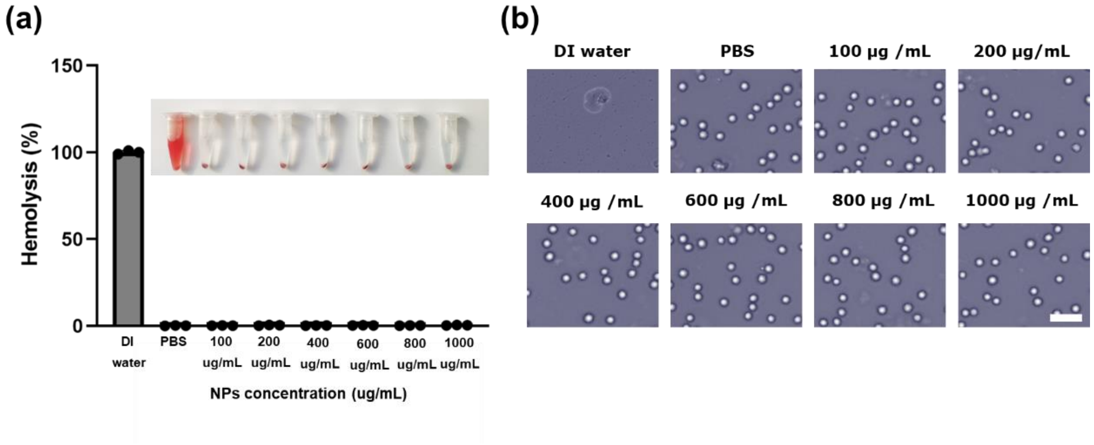

Figure S22. (a) Hemolysis of B1L@SpAcDex-ATMO-21 NPs solution at various concentrations. Inset: the mixtures were centrifuged to detect the presence of hemoglobin in the supernatants visually. (b) The morphology of red blood cells treated with B1L@SpAcDex-ATMO-21 NPs at different concentrations. Scale bar: 50 μm Data are presented as means ± SD (n = 3).

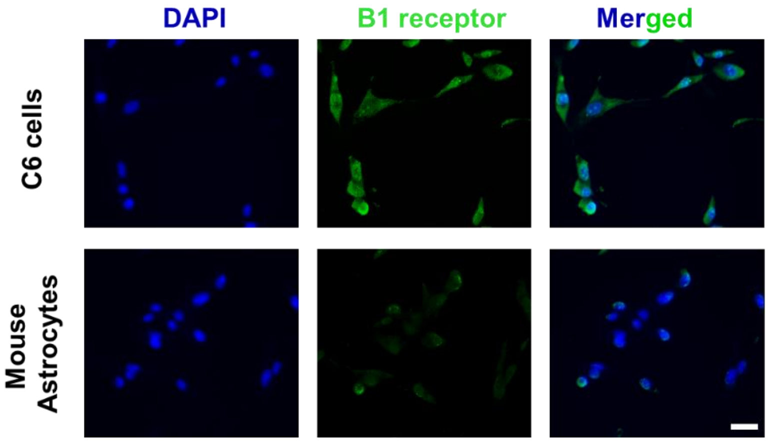

Figure S23. The expression of the B1 receptor (green fluorescence) on C6 cells and mouse astrocytes visualized by CLSM. Scale bar, 20 μm

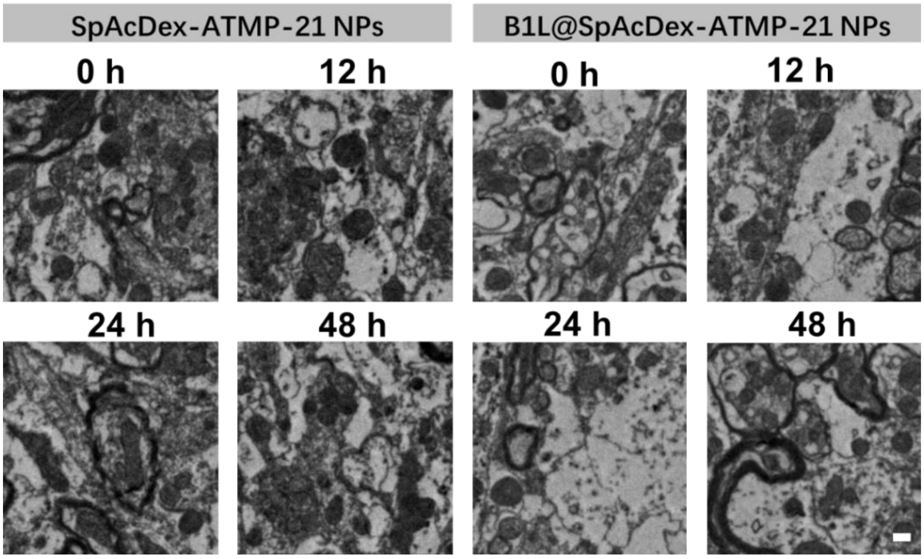

Figure S24. Bio-TEM images of brain blood vessels strctures after and SpAcDex-ATMO-21 NPs and B1L@SpAcDex-ATMO-21 NPs injection at different time point (0, 12, 24, and 48 h). Scale bar, 1 μm

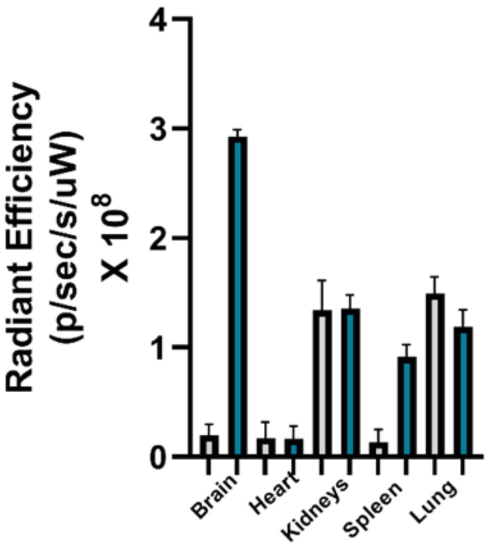

Figure S25. Semiquantitative fuorescence analysis of major organs after injection. Data are presented as means ± SD (n = 3).

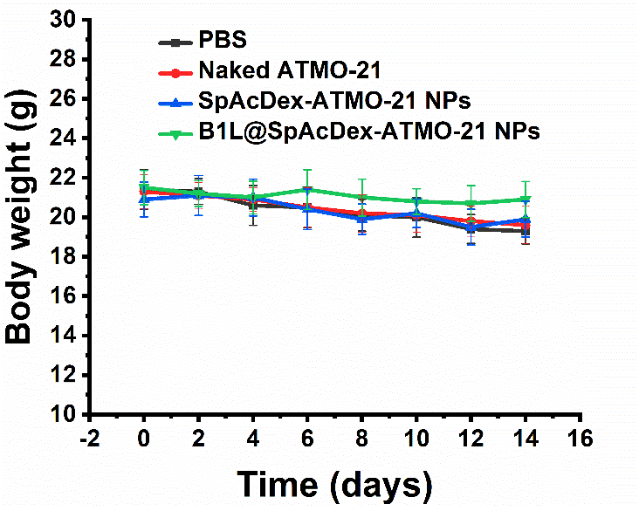

Figure S26. Body weight changes of orthotopic brain tumour-bearing mice of different groups with different treatments. Data are presented as means ± SD (n = 3).

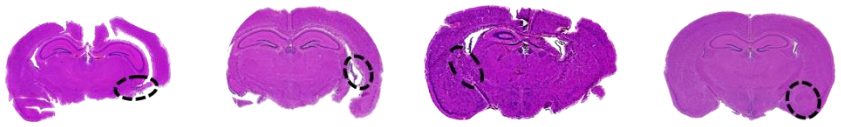

Figure S27. Representative H&E stained images of brain tumour, which were collected from the mice after different treatments.

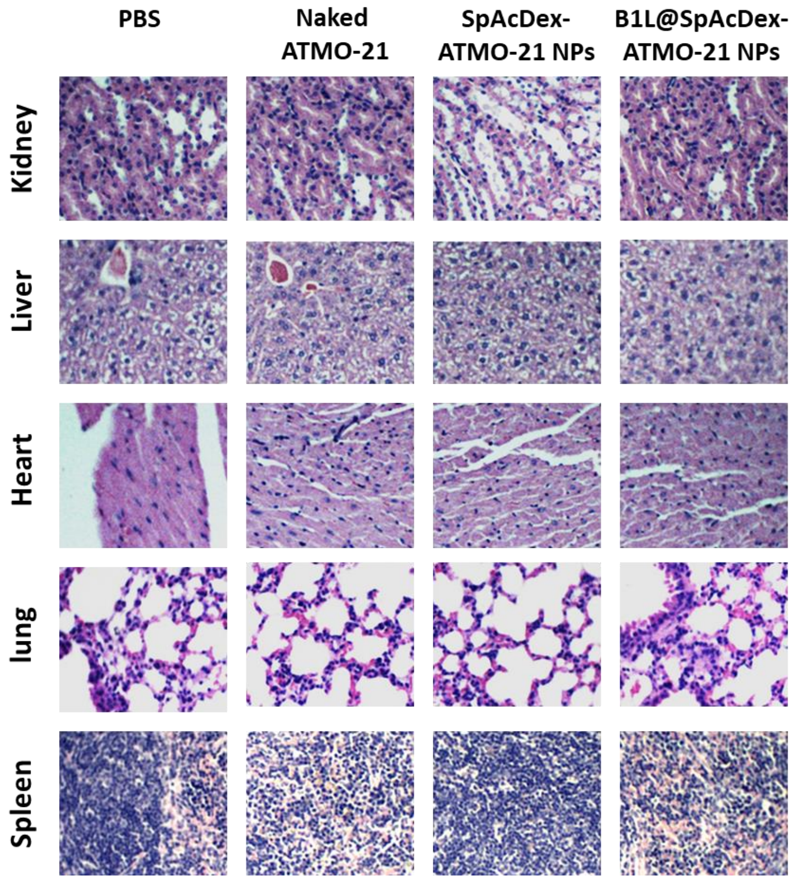

Figure S28. Representative H&E stained images of major organs, which were collected from the mice after different treatments.

3. Reference

[1] G. Kim, M. Kim, Y. Lee, J. W. Byun, D. W. Hwang, M. Lee, *J. Control. Release* **2020**, *317*, 273.
[2] J. L. Cohen, S. Schubert, P. R. Wich, L. Cui, J. A. Cohen, J. L. Mynar, J. M. J. Fréchet, *Bioconjug. Chem.* **2011,** 22, 1056.
[3] N. N. Parayath, S. B. Stephan, A. L. Koehne, P. S. Nelson, M. T. Stephan, *Nat. Commun.* **2020,** 11.
[4] Y. Fan, Y. Cui, W. Hao, M. Chen, Q. Liu, Y. Wang, M. Yang, Z. Li, W. Gong, S. Song, Y. Yang, C. Gao, *Bioact. Mater.* **2021,** 6, 4402.
